# Development and validation of a long-term co-maturation protocol for human stem cell-derived microglia and neuronal networks

**DOI:** 10.1016/j.stemcr.2026.103053

**Published:** 2026-08-20

**Authors:** Annika Mordelt, Imke M.E. Schuurmans, Nicky Scheefhals, Marina P. Hommersom, Koen Slottje, Kimberly Mast, Mara Graziani, Carlos O. González, Klaas W. Mulder, Laura J.A. Wingens, Dirk Schubert, Nael Nadif Kasri, Lot D. de Witte

**Affiliations:** 1 Radboud University Medical Center, Department of Human Genetics, Nijmegen, the Netherlands; 2 Donders Institute for Brain, Cognition and Behaviour, Medical Neuroscience Department, Nijmegen, the Netherlands; 3 Department of Molecular Developmental Biology, Radboud Single Cell Center, Radboud University, Nijmegen, the Netherlands; 4 Radboud University Medical Center, Department of Psychiatry, Nijmegen, the Netherlands

**Keywords:** microglia, neurons, iPSC, differentiation, protocol, co-culture, long-term, neuron-microglia communications, neuro-immune interactions

## Abstract

Microglia-neuron interactions play a key role in a variety of central nervous system disorders. Technologies using human induced pluripotent stem cells (hiPSCs) have been developed to model human brain cells with the goal to understand their function. To effectively study neuro-immune crosstalk and investigate microglial contributions to neuronal network development and function, both microglia and neurons should co-mature allowing for long-term interactions throughout their differentiation. Here, we present a co-maturation protocol that robustly generates glutamatergic neuronal networks containing hiPSC-derived microglia. We validated the long-term co-cultures using single-cell transcriptomics, imaging, and neuronal activity readouts.

In this protocol, astrocytes were required for long-term survival of microglia and for their integration into neuronal networks. Our co-maturation approach induced the typical ramified microglia morphology and characteristic microglia-neuron interactions. Homeostatic markers such as P2RY12 and TMEM119 and neuronal remodeling-associated genes were upregulated compared to microglia monocultures, highlighting the necessity of the environment to generate and maintain the context-dependent microglia signature *in vitro*. In this manuscript, we include the full optimization process of our co-maturation approach, a comprehensive description of the protocol, practical guidelines, and troubleshooting tips. Our co-maturation model provides a powerful tool to assess the role of human microglia in modulating neuronal function and development in health and disease.

## Highlights

• hiPSC protocol enables co-maturation of microglia and Ngn2-induced neuronal networks
• Astrocytes are required for long-term microglial survival and network integration
• Co-maturation induces microglial signature on morphology, transcriptome and protein level

## Teaser

In this article, Mordelt and colleagues present a hiPSC-based protocol generating microglia within glutamatergic neuronal networks. Their long-term co-maturation approach enhances microglial ramification and homeostatic marker expression and enables dynamic neuron-microglia interactions. The method, validated using single-cell transcriptomics, imaging, and electrophysiological assays, offers a robust tool to study human microglia in neuronal development and disease.

## Introduction

Neuro-immune interactions are increasingly recognized to be critical for brain function in health and disease. Microglia, the tissue-resident immune cells of the brain, are at the intersection of neuronal and immunological activity in the central nervous system (CNS) (Borst et al., 2021; Nayak et al., 2014). By continuously surveilling the CNS, microglia shape and control neuronal circuits (Umpierre and Wu 2021; Schafer et al., 2012; Cheadle et al., 2020). Beside the more classical myeloid functions in response to danger signals, such as initiating inflammatory responses and phagocytosis, the continuous and dynamic interaction with neurons is thought to be central to microglial function and the CNS. Mechanisms such as synaptic remodeling have been repeatedly highlighted in the field for the last decade (Paolicelli et al. 2011, 2022; Prinz et al., 2019; Eyo et al., 2021), and are speculated to be a major mechanism involved in neurodevelopmental, neuroinflammatory, and neurodegenerative disorders (Salter and Stevens 2017; Priller and Prinz 2019; Colonna and Butovsky 2017; Zengeler and Lukens 2021). Therefore, there is an ongoing effort to understand microglial function, microglia-neuron crosstalk, and its role in CNS disease.

Unraveling these processes is challenged by the following factors: (1) microglia adopt diverse states, and their function is context dependent (Dubbelaar et al., 2018; Depp et al., 2025; Matcovitch-Natan et al., 2016; Gosselin et al., 2017), so we need models that recapitulate the *in vivo* microglia phenotypes. (2) Microglia function and glia-neuron communication are species specific (Gosselin et al., 2017; Lloyd et al., 2024), so we need human models to complement rodent *in vivo* models. (3) Microglia-neuron interactions are fast and dynamic, based on their regional context. Models should be able to measure microglia-neuron interactions over time. (4) These interactions take place at the subcellular level. Models should offer the resolution to analyze neuron-microglia interactions at the level of dendrites and synapses. (5) The ultimate output of these interactions is neuronal function. We should build models that can measure the impact of neuron-microglia interactions on neuronal network activity. Current models, including microglial cell lines, postmortem tissue, rodent models, and human induced pluripotent stem cell (hiPSC) models, have been valuable to the field, with their own benefits and challenges. To complement these models, we optimized a hiPSC-derived neuron-microglia co-maturation model that addresses all of the five challenges described above.

The discovery of induced pluripotent stem cells (iPSCs) (Takahashi and Yamanaka 2006) and their use for *in vitro* modeling of CNS disorders yielded opportunities for the neuroimmunology field. Many advances in human microglia modeling have been made (Hanger et al., 2020; Jäntti et al., 2024). Still, difficulties in maintaining the microglia signature *in vitro* remain. Without continuous input of the brain environment, microglia rapidly lose their characteristic phenotype, including a downregulated expression of key markers and a change of their morphology (Gosselin et al., 2017; Holtman et al., 2017). This environment-dependent nature of microglia poses significant challenges for accurate *in vitro* modeling. As a result, monoculture systems, whether based on primary microglia or those derived from hiPSCs, are limited in their ability to replicate the physiological properties of microglia. Further, interactions with neurons are fundamental to microglial physiology and pathology (Paolicelli et al., 2022; Prinz et al., 2019; Salter and Stevens 2017). Investigating how microglia regulate neuronal circuits requires models that recapitulate the dynamic and continuous interplay between both cell types. To tackle this, microglia-neuron co-cultures or more complex organoid models (sometimes xenografted into rodent brains) have been developed in the last decade (Jäntti et al., 2024; Schafer et al., 2023), with each approach offering distinct advantages and limitations. Some co-culture strategies employ separate differentiation of microglia and neurons, followed by a brief co-culture of 24 h (Mathews et al., 2023; Dräger et al., 2022) or 3–6 days (Lish et al., 2025), while another protocol reported a longer co-culture duration of 14 days (Ryan et al., 2020). It is clear that the iPSC field is expanding to study cell-cell interactions and their effects on human brain function. To add to this effort, we developed and validated a human iPSC-based 2D model that provides advantages for studying microglia-neuron crosstalk such as high temporal and spatial resolution, possibilities to manipulate and measure at both cellular and subcellular level, and longitudinal non-invasive recording of neuronal network function (Mossink et al., 2021).

A widely used 2D model to study neuronal function is *Neurogenin-2* (*Ngn2*)-induced glutamatergic neurons (Zhang et al., 2013), which are routinely cultured with embryonic (E18) rat astrocytes to support neuronal health and maturation (Mossink et al., 2021; Frega et al., 2017; van Hugte et al., 2023). Their rapid differentiation and synapse formation make them well suited for activity assays and disease phenotyping (Mossink et al., 2021; van Hugte et al., 2023). Here, we present a fast and robust hiPSC-based protocol that allows for the co-maturation of microglia and with *Ngn2*-induced neurons. This manuscript describes the full optimization process and offers practical guidelines. Our co-maturation protocol enables sustained interaction throughout development and long-term co-culturing of both cell types while preserving cell-type identities. The protocol promotes characteristic microglia-neuron interactions and induces the typical ramified microglia morphology and expression of microglial markers, underscoring the need for the presence of neurons to study microglial function.

## Results

**Figure 1:**
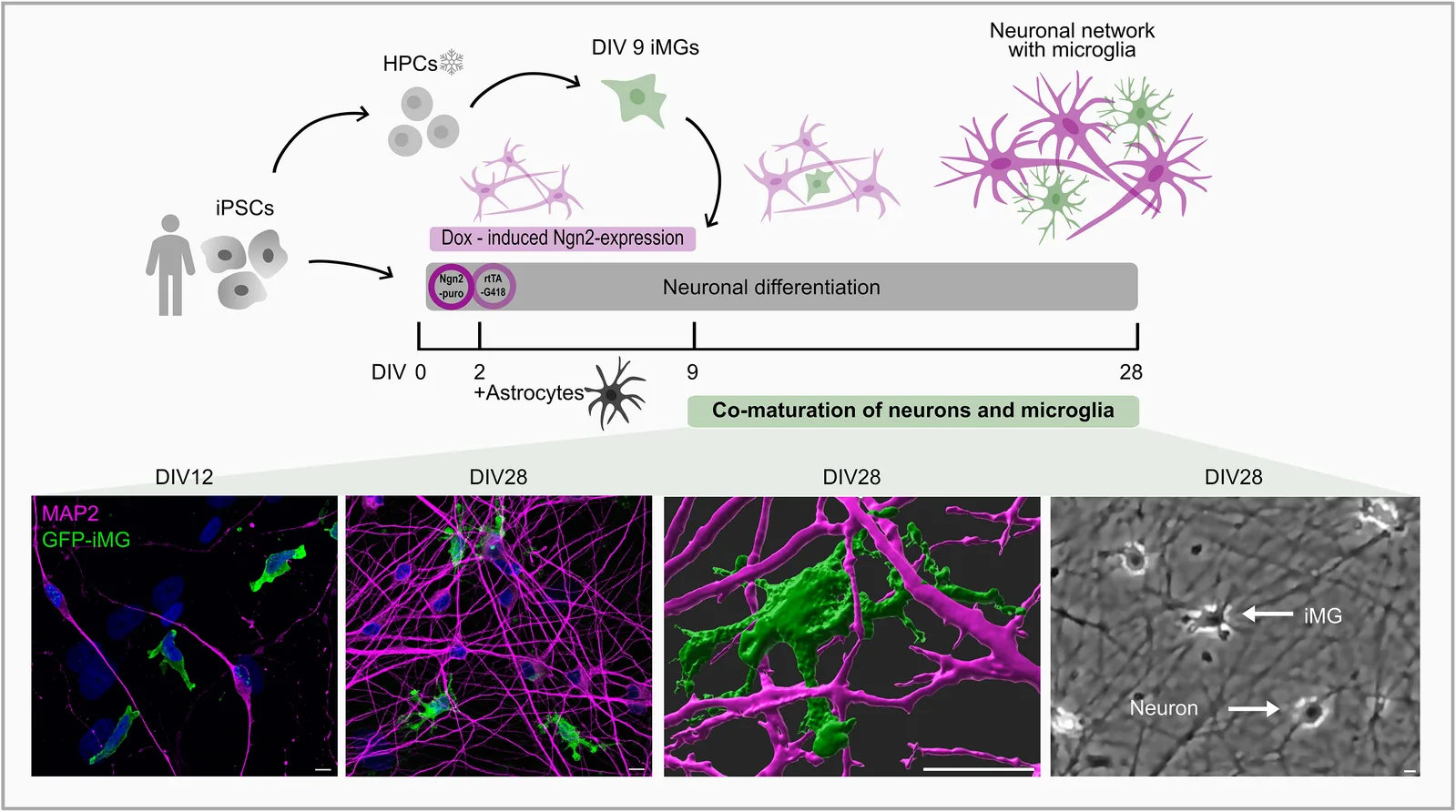
HiPSC-based protocol allowing for co-maturation of microglia and neuronal networks Schematic protocol overview for co-maturation cultures of hiPSC-derived microglia (iMGs) and iNeurons. DIV (days *in vitro*) 9 iMGs derived from hematopoietic progenitor cells (HPCs) are added to *Neurogenin2* (*Ngn2*)-induced neuronal networks cultured with rat astrocytes after withdrawal of doxycycline and co-mature together in the same medium (top). Representative fluorescence microscope images show developing iMGs (green) and neurons (magenta) at DIV 12 and DIV 28 (bottom left) and DIV 28, and digital reconstruction and brightfield image of iMG in neuronal network at DIV 28 (bottom right); scale bars of all four images, 10 μm.

We developed a co-maturation model where both hiPSC-derived microglia (iMGs) and *Ngn2*-induced neurons (iNeurons) (Zhang et al., 2013; Frega et al., 2017) differentiated alongside each other (Figure 1). In our model, iMGs were added to the neurons at days *in vitro* (DIV) 9, since this is the time point where neuronal lineage induction was stable and doxycycline could be withdrawn to minimize side effects on microglia differentiation. From DIV 9 to DIV 28, iMGs and neurons co-matured together and developed their characteristic ramifications and dendrites, respectively (Figure 1). The iMGs integrated into the dense neuronal networks and formed contact sites with the dendrites. We tested the protocol using six hiPSC lines for iMG differentiation across researchers and laboratories (Figure S1) to ensure generalizability and robustness of the protocol. In addition to a comprehensive description of the co-maturation protocol in the STAR Methods section, recommendations and troubleshooting tips can be found in Table S1. Below, we describe in detail the steps and considerations taken to develop and validate this co-maturation protocol.

### Defining a neuron-compatible medium for microglia differentiation

**Figure 2:**
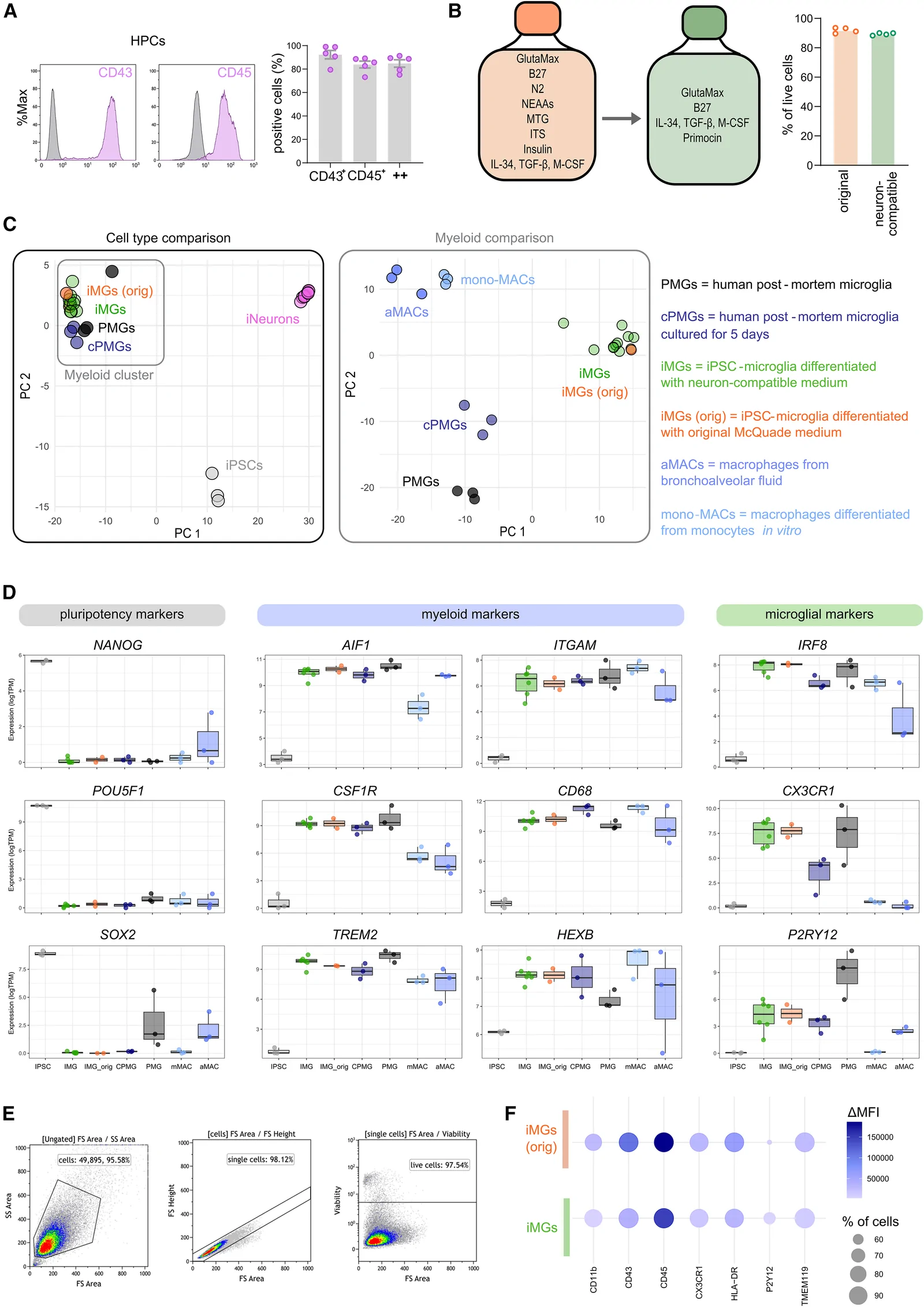
Benchmarking of iMGs differentiated with neuron-compatible medium (A) Flow cytometry of hematopoietic progenitor cells (HPCs) at DIV (days *in vitro*) 11 showing representative CD43 and CD45 expression (left) and quantification of percentage of positive cells, *N* = 5 (right). (B) Medium composition of identified neuron-compatible medium for iMG differentiation (left) and live-cell count comparison of medium compositions using flow cytometry, *n* = 4 replicates (right). (C) Principal-component analysis (PCA) of gene expression profiles of different cell types, based on the top 500 variable genes, *N* = 3–10 independent replicates (iPSCs, iMGs, iNeurons) or different donors (PMGs, cPMGs, aMACs, mono-MACs) per cell type; all samples were sequenced in the same batch. (D) Boxplots showing gene expression as transcripts per million (TPM) of key pluripotency, myeloid, and microglial markers per cell type. (E) Flow cytometry gating strategy for iMGs. (F) Bubble blot showing proteins measured with flow cytometry in iMGs differentiated with the two different media. MFI, mean fluorescence intensity; delta was calculated by subtracting the MFI of the unstained sample; % of cells = percentage of live cells positive for corresponding antibody. iMGs for data in this figure were generated from GM25256 (Coriell Institute) and AICS-0016, cl.18 (Allen Institute).

Co-maturation of iMGs and neurons required the identification of a medium composition that supports microglial lineage commitment while remaining compatible with *Ngn2*-induced neuronal differentiation. We began by adapting the protocol established by McQuade et al., (2018), which involves the stepwise differentiation of iMGs via hematopoietic progenitor cells (HPCs). We generated HPCs and confirmed their identity by detecting the expression of canonical markers CD43 and CD45 using flow cytometry (Figure 2A).

To optimize HPC harvesting, we analyzed the cultures from DIV 8 to DIV 12 (Figure S2A). We measured the expression of CD206, a marker of differentiation into brain-associated macrophages, which microglial precursors do not express (Utz et al., 2020). The percentage of CD206 expressing HPCs increased from 11% at DIV 11 to 20% at DIV 12 (Figure S2B). To reduce variability across cells and batches, we restrained from multiple harvest days as described in the original protocol and selected DIV 11 as the standardized harvesting time point. Additionally, we successfully introduced a cryopreservation step to enable batch-wise HPC production, minimize variability, and streamline downstream workflows.

Next, we screened medium components using an all-minus-one fashion to identify the essential medium components for iMG differentiation from the HPC stage. We identified a simplified and neuron-compatible version of the original McQuade medium omitting N2, NEAAs, MTG, ITS, and insulin (Figures 2B and S3A; see STAR Methods). Beside the essential microglial growth cytokines (IL-34, TGF-β, and M-CSF) the remaining components are shared with the *Ngn2*-induced neuronal differentiation (Frega et al., 2017), and this neuron-compatible medium preserved cell viability (Figure 2B).

### Neuron-compatible medium induces gene, protein, and functional profile of microglia

We next examined the molecular phenotypes induced by our neuron-compatible iMG differentiation. To do this, we compared the transcriptomes of iMGs differentiated in the neuron-compatible medium to primary human postmortem-derived microglia, cultured for 5 days (CPMG) or non-cultured (PMG), monocyte-derived macrophages (mMACs), alveolar macrophages (aMACs), hiPSCs, and hiPSC-derived neurons. We processed and sequenced these different cell types in the same batch as the iMGs to minimize batch effects.

To benchmark in comparison to existing approaches, we differentiated iMGs using the original medium from McQuade et al. (2018). Principal component analysis showed that iMGs clustered with other microglia types and away from hiPSCs, indicating that the neuron-compatible medium successfully induces a microglia-like transcriptional profile (Figure 2C). However, when we focused on the myeloid cells, we found that monoculture iMGs, whether differentiated with our neuron-compatible medium or the original McQuade medium, remained distinct from postmortem microglia (Figure 2C).

We next analyzed the expression of key genes to evaluate microglial identity (Figure 2D). First, we confirmed that pluripotency markers were most highly expressed in hiPSCs and absent in iMGs (Figure 2D). Myeloid markers such as *AIF1*, *ITGAM*, *CD68*, and *HEXB* showed markedly higher expression in iMGs, cPMGs, PMGs, mMACs, and aMACs, in contrast to hiPSCs, as expected. *CSF1R* and *TREM2* were more strongly expressed in iMGs, cPMGs, and PMGs than in mMACs or aMACs (Figure 2D). *CX3CR1* expression was restricted to iMGs, cPMGs, and PMGs and was not detected in mMACs and aMACs. Notably, *P2RY12* showed high expression in PMGs and was lower when the PMGs were cultured (cPMGs) and in iMGs (Figure 2D). This is consistent with previous findings that highlight the sensitivity of homeostatic marker expression to environmental context (Schafer et al., 2023). These observations support the notion that monoculture conditions fail to induce a fully homeostatic microglial gene signature (Gosselin et al., 2017) and emphasize the importance of incorporating neuronal and other CNS-resident cells to study microglia *in vitro*. Importantly, all genes analyzed showed highly similar expression levels across the two media conditions used for iMG differentiation, indicating that the neuron-compatible medium induces a microglial identity comparable to that achieved with the original McQuade medium at the gene expression level (Figure 2D).

Since transcript levels do not always correspond to protein abundance (Lloyd et al., 2024; Johnson et al., 2022), we next compared protein levels of key proteins in iMGs differentiated with the neuron-compatible and the original McQuade medium using flow cytometry (Figures 2E, 2F, S3B, and S3C). Our analysis revealed that both the proportion of cells expressing CD11b, CX3CR1, HLA-DR, and TMEM119 as well as their fluorescence intensities were highly comparable between the two media conditions (Figure 2F). CD43 and CD45 expression levels were slightly reduced, and a greater proportion of iMGs exhibited low P2RY12 expression when differentiated in the neuron-compatible medium (Figure 2F). We consistently detected CD45 and CX3CR1 co-expression in iMGs differentiated with the neuron-compatible medium across replicates using flow cytometry (Figure S3D) and CX3CR1, TREM2, P2RY12, and TMEM119 expression across hiPSC lines using immunocytochemistry (Figures S4A and S4B).

To validate microglial functionality, we assessed phagocytosis and confirmed internalization of pHrodo-labeled particles using live-cell imaging (Figures S4C and S4D). We further tested the iMGs’ ability to respond to inflammatory stimuli by analyzing protein secretion profiles in response to stimulation with hydrocortisone, ATP, IFN-γ, and TNF-α using Olink proteomics. This analysis revealed cell-type-specific baseline signatures and stimulus-specific protein responses (Figures S4E and S4F). Together, these findings validated that our neuron-compatible medium induces characteristic gene expression, protein profiles, and functional behaviors of microglia. We concluded that this formulation yields a robust monoculture platform comparable to established iMG differentiation protocols and offered a strong basis for the subsequent co-maturation protocol with neurons.

### Astrocytes are required for long-term microglia survival and integration into neuronal networks

**Figure 3:**
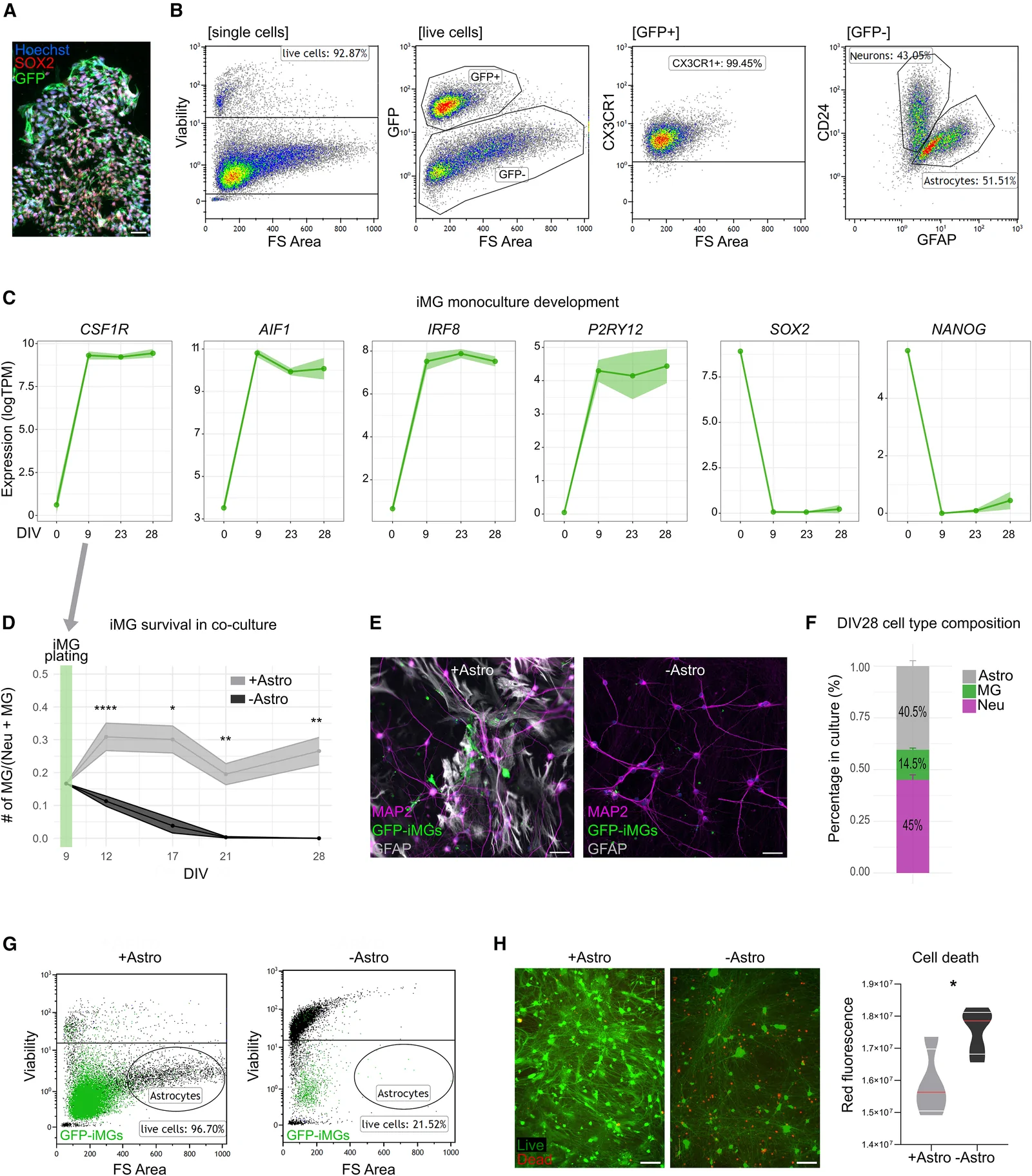
Astrocytes are required for long-term survival and integration into neuronal networks (A) Representative fluorescence microscope image of GFP-tagged hiPSC colony; scale bar, 50 μm. (B) Flow cytometry panels showing gating strategy for the detection of GFP-positive microglia in co-cultures. (C) Gene expression of myeloid and pluripotency markers across developmental timeline of iMG differentiation in monoculture, TPM, transcripts per million; *N* = 3 for DIV0 and DIV23, *N* = 2 for DIV (days *in vitro*) 9 and DIV 28. (D) Quantification of iMG numbers in cultures across development, *N* = 2, *n* = 2,102 neurons for +Astro, *n* = 1,854 neurons for −Astro; data represent mean ± SEM, ^∗^*p* < 0.05, ^∗∗^*p* < 0.005, ^∗∗∗∗^*p* < 0.0001. (E) Representative fluorescence microscope images of cultures with (+Astro) or without (−Astro) astrocytes at DIV 28; scale bars, 50 μm. (F) Cell-type composition in cultures at DIV 28 quantified by immunocytochemistry images; *N* = 2, *n* = 932 cells; data represent mean ± SEM. (G) Flow cytometry panels showing GFP-iMGs and live-cell percentages in co-cultures with or without astrocytes. (H) Representative fluorescence images of live-dead staining in co-cultures with or without astrocytes; scale bars: 100 μm (left); and quantification of cell death at DIV 22 using the integrated density of the red fluorescence; *N* = 1, *n* = 5 cultures per condition, ^∗^*p* < 0.05 (right). iMGs for data in this figure were generated from AICS-0016; iNeurons were generated from GM25256.

After optimizing the neuron-compatible medium for iMGs in monoculture, we proceeded to develop the co-maturation protocol. To support neuronal differentiation, we supplemented the medium with the growth factors NT3 and BDNF, which are not required for microglial differentiation in monoculture. We replaced DMEM/F12 with Neurobasal, which is optimized for neuronal health and activity, while retaining all other components from the neuron-compatible iMG medium (see STAR Methods). To facilitate monitoring of microglia in various conditions, we used a GFP-actin-tagged hiPSC line to generate fluorescently labeled iMGs (Figures 3A and 3B).

We introduced DIV 9 iMGs into the neuronal culture (Figure 1), as they already expressed key microglial genes such as *CSF1R*, *AIF1*, *IRF8*, and *P2RY12* at this stage, while pluripotency markers *SOX2* and *NANOG* were no longer detectable (Figure 3C), indicating successful induction of the myeloid lineage from the hiPSC state. To compare the developmental maturity of the iMGs to public data, we assessed juvenile and adult microglial signatures in iMGs and PMGs (Figure S8A). The data suggest that, while iMGs adopt a microglial lineage, they remain transcriptionally less mature than PMGs, likely due to the absence of environmental cues. The time point DIV 9 was therefore optimal for introducing iMGs into the neuronal cultures, because (1) iMG differentiation was still at an early stage, providing sufficient time for iMGs to undergo plastic changes upon interaction with neurons enhancing their maturation; (2) the initial wave of proliferation had already occurred, allowing for better control over iMG numbers; and (3) pluripotent markers were absent.

During co-culture protocol optimization, we identified astrocytes as essential for establishing a stable long-term co-culture. Within the neuronal culturing field, it is standard practice to add rodent *ex vivo* astrocytes to promote healthy neuronal differentiation and function *in vitro* (Zhang et al., 2013; Frega et al., 2017; Banker 1980). When we omitted astrocytes at DIV 2, microglial numbers declined rapidly and were undetectable by DIV 21 (Figures 3D and 3E). In contrast, when we added astrocytes, microglial numbers remained stable from DIV 10 to DIV 28 (Figure 3D). While most of our experiments were performed at DIV 28, we could maintain the co-maturation culture until DIV 51 (Figure S1). Next, we characterized the cell-type composition at DIV 28. Because techniques such as flow cytometry and single-cell RNA sequencing (RNA-seq) introduce biases in cell-type ratios, due to the difficulty in dissociating dense neuronal networks, we opted for immunocytochemistry-based quantification (Figure 3E) and only relied on flow cytometry measurements when comparing conditions within a cell type. Neurons comprised approximately 45% of the cultures, microglia 15%, and astrocytes the remaining 40% (Figure 3F).

We hypothesized that the microglia loss in the absence of astrocytes resulted from either impaired integration into the network or increased cell death. To test this, we performed flow cytometry and observed a drastic reduction of live-cell counts in the condition without astrocytes (96%–21%) accompanied by a reduction of GFP-iMGs (Figure 3G). Additional live-cell imaging confirmed increased cell death when astrocytes were not present (Figure 3H). Since neuronal numbers remained stable from DIV 12 to DIV 28 when cultured without astrocytes (Figure S5A), the observed cell death could be attributed to the microglia. Next, we quantified microglial integration by comparing microglial numbers in the culture supernatant and the adherent fraction after dissociation using flow cytometry. The presence of astrocytes increased microglial integration from ∼10% to 97% (Figure S5B), indicating that astrocytes promote both microglial survival and integration. We speculated that toxic glutamate accumulation in neuronal networks due to the lack of astrocytic clearance could be driving this effect. Indeed, glutamate levels were higher in the supernatant of co-cultures without astrocytes (Figure S5C). We pharmacologically blocked excitatory amino acid transporters (EAATs) with DL-TBOA (DL-threo-β-benzyloxyaspartate) in co-cultures with astrocytes leading to an increase of glutamate to the level of the −Astro condition (Figure S5C). However, treatment with DL-TBOA did not mimic the decrease in viability observed in co-cultures without astrocytes (Figure 3J), suggesting that alternative mechanisms underlie the astrocyte-dependent support.

### Co-maturation protocol induces signature morphology, transcriptome, and proteins of human microglia

**Figure 4:**
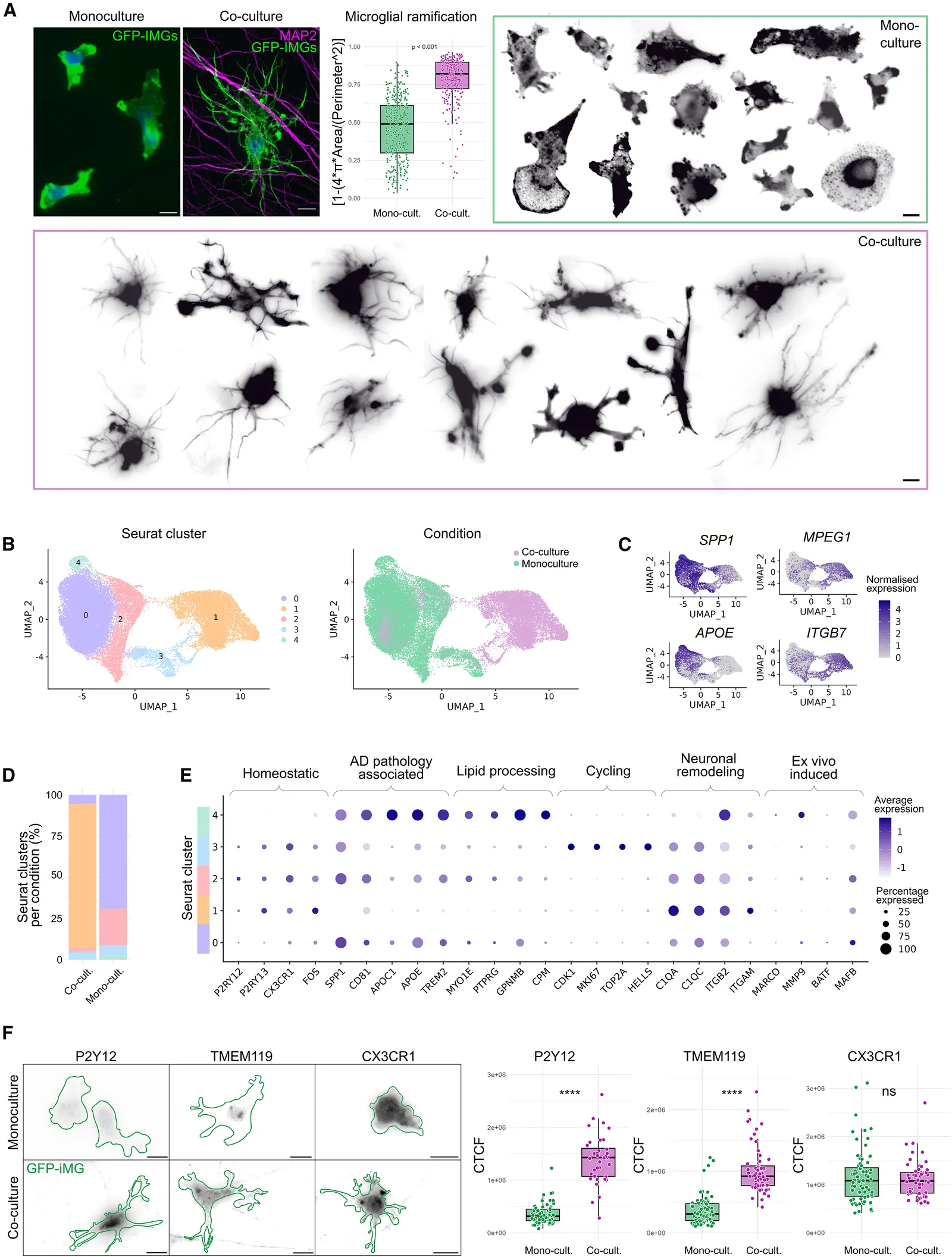
Morphological, transcriptomic, and protein expression changes of iMGs in the co-maturation protocol compared to monoculture (A) Representative fluorescence microscope images of GFP-actin based iMG morphology in monoculture vs. the co-culture with neurons at DIV (days *in vitro*) 28. Scale bars: 10 μm (top left). Quantification of ramification index per cell, *N* = 3, *n* = 352 cells for monoculture, *n* = 249 cells for co-culture, unpaired *t* test (top middle), and grayscale-inverted images of iMGs per culture condition. Scale bars: 10 μm (top right and bottom). (B) Uniform manifold approximation and projection (UMAP) analysis of single-cell RNA-seq data of iMGs at DIV 28 showing Seurat clusters (left) and the culture condition (right); *N* = 3, *n* = 42,226 single cells. (C) UMAP analysis showing top differentially expressed genes of co-culture vs. monoculture iMGs (average log2 fold changes are *SPP1* = −4.35, *APOE* = −4.29, *MPEG1* = 2.99, *ITGB7* = 2.9, and all show *p* < 0.0001). (D) Distribution of Seurat clusters per culture condition. (E) Average gene expression per Seurat cluster highlighting signatures associated with different microglia contexts. (F) Representative fluorescence microscope images of P2RY12, TMEM119, and CX3CR1 protein expression in iMGs in monoculture vs. co-culture; cell outline determined by GFP-actin signal. Scale bars, 10 μm (left) and quantification of fluorescence signal per cell; CTCF, corrected total cell fluorescence, *N* = 2, P2RY12; *n* = 59 cells for monoculture, *n* = 36 cells for co-culture, TMEM119; *n* = 68 cells for monoculture, *n* = 57 cells for co-culture, CX3CR1; *n* = 80 cells for monoculture, *n* = 55 cells for co-culture, unpaired *t* test, ^∗∗∗∗^*p* < 0.0001. iMGs for data in this figure were generated from AICS-0016; iNeurons were generated from GM25256.

Microglia undergo rapid changes in gene expression, protein levels, and morphology when they are removed from the CNS (Lloyd et al., 2024; Holtman et al., 2017). Vice versa, co-culture and xenotransplant models have shown that the interaction with CNS-resident cells induces typical microglia features (Schafer et al., 2023; Dräger et al., 2022). Given the continuous interactions between iMGs and neurons in our culture model, we speculated that the co-maturation approach may support microglia identity. We therefore assessed the impact of our co-culture on iMG morphology and gene and protein expression. First, we examined iMG ramification in the neuron-glia networks (co-culture) compared to iMG monocultures. Immunocytochemistry analysis of GFP-tagged iMGs showed a significant increase in ramification in the co-culture condition (Figure 4A). We observed high levels of morphological heterogeneity in both conditions but a robust effect on ramification by the presence of neuron-glia networks (see representative microscopy images in Figure 4A).

To assess the effect of the co-maturation at the transcriptomic level, we compared iMGs from both culture conditions using single-cell RNA-seq. In this experiment, the initial starting vials of HPCs, coating, medium master mixes, and cell culture schedule were identical to ensure the presence of neuron-glia networks is the only variable factor. Unsupervised clustering revealed a clear distinction between the two conditions (Figure 4B), indicating that the neuron-glia network environment substantially changed gene expression in iMGs. Using differential analysis, we identified *SPP1* and *APOE* as the top downregulated genes in co-culture iMGs compared to monoculture iMGs (log fold change below −4). Interestingly, these are the top two contributors to the disease-associated microglia signature often observed in Alzheimer’s disease (AD) pathology (Lan et al., 2024). Among the top upregulated genes in co-culture iMGs were *MPEG1* and *ITGB7*, which is coding for an integrin protein involved in cell-cell adhesion (Figure 4C). Next, we investigated characteristic gene sets of microglia substates and functions based on transcriptional studies of human postmortem microglia (Holtman et al., 2017; Patir et al., 2019; Sun et al., 2023; Masuda et al., 2019), the environment-dependent signature described by Gosselin et al., (2017), and the current consensus in microglia nomenclature (Paolicelli et al., 2022). Cluster 1, where the majority of co-culture iMGs were distributed in Figure 4D, showed an upregulation of homeostatic and neuronal remodeling markers (Figure 4E). In contrast, genes associated with AD pathology and lipid processing were strongly downregulated compared to all other clusters. The most prominent cluster in the monoculture iMGs (cluster 0) showed low expression for the homeostatic genes. Cluster 3 was identified as cycling microglia and contained both monoculture and co-culture iMGs. Cluster 4 comprises a small subset of monoculture iMGs with highest expression of AD pathology-related genes and no homeostatic signature gene expression (Figure 4E). Genes associated with the *ex vivo* signature induced by culturing of postmortem microglia (Gosselin et al., 2017) were expressed lower in co-culture iMGs (cluster 1) compared to monoculture iMGs (clusters 0, 2, 4) (Figure 4E). This suggests that the co-culture environment partially buffers the effects observed when microglia are cultured without brain context. Further analysis of public gene sets describing the adult microglial signature (Galatro et al., 2017) revealed substantial overlap with the top upregulated genes in the co-culture iMGs compared to monoculture iMGs (such as *MPEG1*; Figure S8B), indicating a shift toward more mature microglia phenotypes in our co-maturation model.

Next, we assessed a panel of key microglia markers on protein level using immunocytochemistry (Figure 4F). Analysis of the fluorescence per cell revealed upregulation of P2RY12 and TMEM119 in co-culture iMGs (Figure 4F). CX3CR1 expression remained unchanged, consistent with its already elevated RNA levels in monoculture iMGs (Figure 2D). In conclusion, the neuron-glia network in our co-maturation model promotes several aspects of *in vivo* microglia identity, highlighting the importance of using long-term co-cultures protocols to model microglia *in vitro*.

### Co-maturation cultures show cell-type-specific profiles and mature neurons with organized synaptic activity

**Figure 5:**
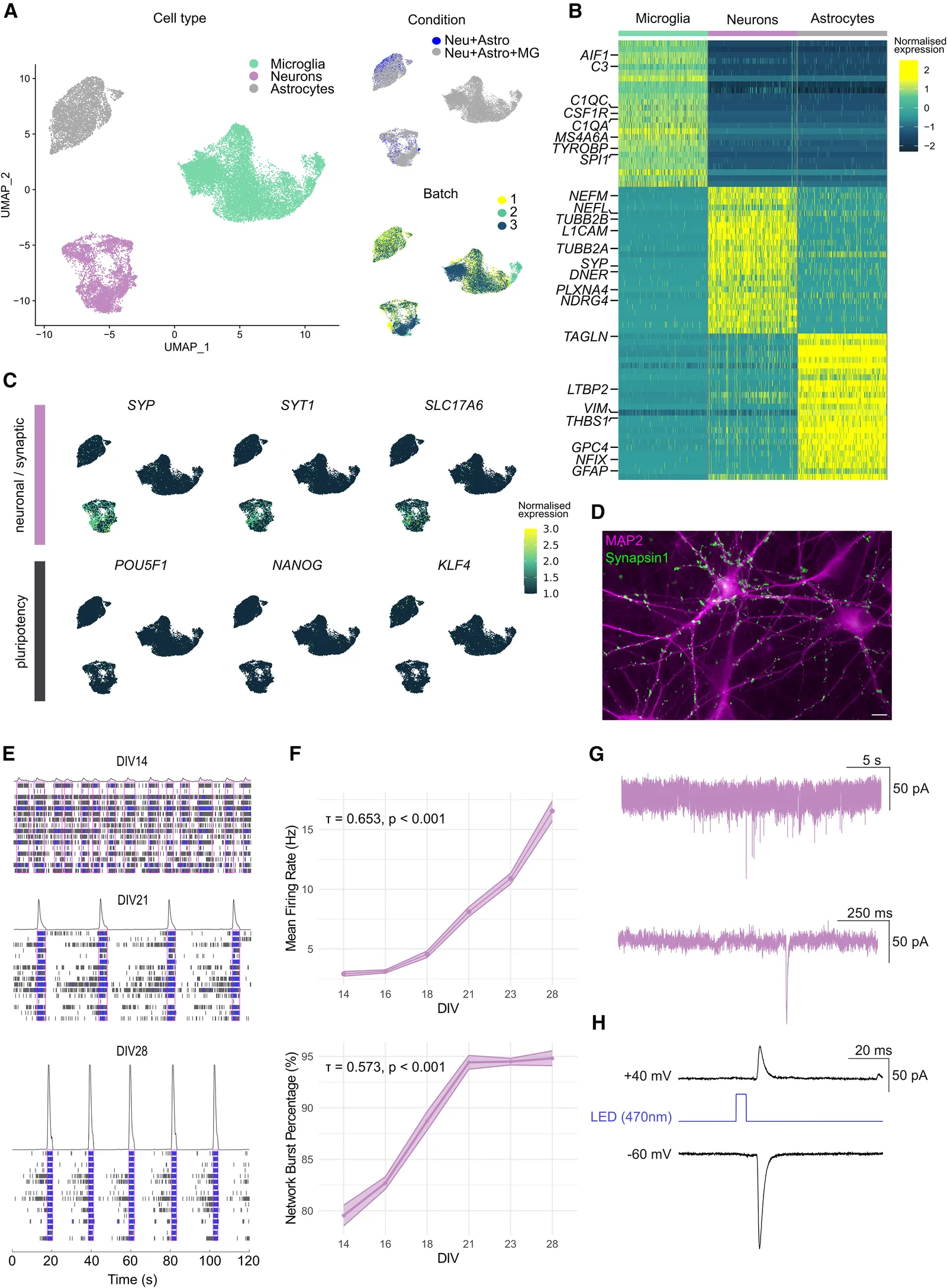
Transcriptional and functional validation of co-maturation cultures (A) Uniform manifold approximation and projection (UMAP) analysis of single-cell RNA-seq data of cultures at DIV (days *in vitro*) 28 showing clustering of the three cell types (left) and distribution of condition and batch (right), *N* = 3, *n* = 24,791 single cells. (B) Heatmap showing expression profile of the top 25 genes per cell type. (C) UMAP analysis showing expression of neuronal (top) and pluripotent (bottom) markers. (D) Representative fluorescence microscopy image of a neuron in the co-maturation culture stained with Synapsin1 (green) and MAP2 (magenta); scale bar, 10 μm. (E) Representative micro-electrode array (MEA) raster plots with network bursts annotated in magenta showing increased organization of neuronal network activity from DIV14 to DIV 28. (F) Quantification of mean firing rate (Hz; top) and network burst percentage (%; bottom) across six developmental time points; *N* = 4, *n* = 81 neuronal networks, data represent means ± SEM, Mann-Kendall test. (G) Representative traces of miniature excitatory postsynaptic currents (mEPSCs) recorded from single neurons at DIV 49 using whole-cell patch-clamp recordings. (H) Traces of light-evoked synaptic currents recorded from a channelrhodopsin-2-negative neuron at DIV 28. iMGs for data in this figure were generated from AICS-0016; iNeurons were generated from GM25256.

To fully characterize the co-maturation cultures on the transcriptome level, we utilized single-cell RNA-seq at DIV 28. Clustering with the Louvain algorithm after Harmony integration revealed three distinct clusters (Figure 5A). Based on signature marker expression of the respective cell types, we annotated microglia, neurons, and astrocytes (Figure 5B). All batches, prepared in separate libraries, showed the presence of all three cell types, and cultures without addition of iMGs contained no cells clustering to the microglia cluster (Figure 5A). The neuronal cluster expressed synaptic markers such as *SYP*, *SYT1*, and *SLC17A6*, indicating lineage commitment to excitatory glutamatergic neurons (Figure 5C). We detected no pluripotent marker expression in any of the three clusters, confirming successful differentiation with no remaining hiPSC colonies in the cultures (Figure 5C). Next, we verified the expression of Synapsin1 using immunocytochemistry, indicating the presence of synapses on the neurons (Figure 5D). To validate the functional neuronal network phenotype of the co-maturation cultures, we recorded spontaneous activity using micro-electrode arrays (MEAs) over time (Figure 5E). We observed an increase in general spiking activity, reflected by the mean firing rate, from DIV 14 to 28 and the synchronization of network activity, reflected by establishment and increase in network burst percentage after DIV14 (Figure 5F). Additionally, on single-neuron level, we verified functional synaptic connectivity in the co-maturation cultures using whole-cell patch-clamp recordings, both by measuring miniature excitatory postsynaptic currents (Figure 5G) and by optogenetically inducing synaptic responses in presynaptic channelrhodopsin2 (ChR2)-expressing neurons (Figures 5H and S6). Taken together, the co-maturation cultures show cell-type-specific transcriptomic profiles and functional properties of neuronal activity on single-cell and network level.

### Co-maturation cultures show key features of microglia-neuron crosstalk

**Figure 6:**
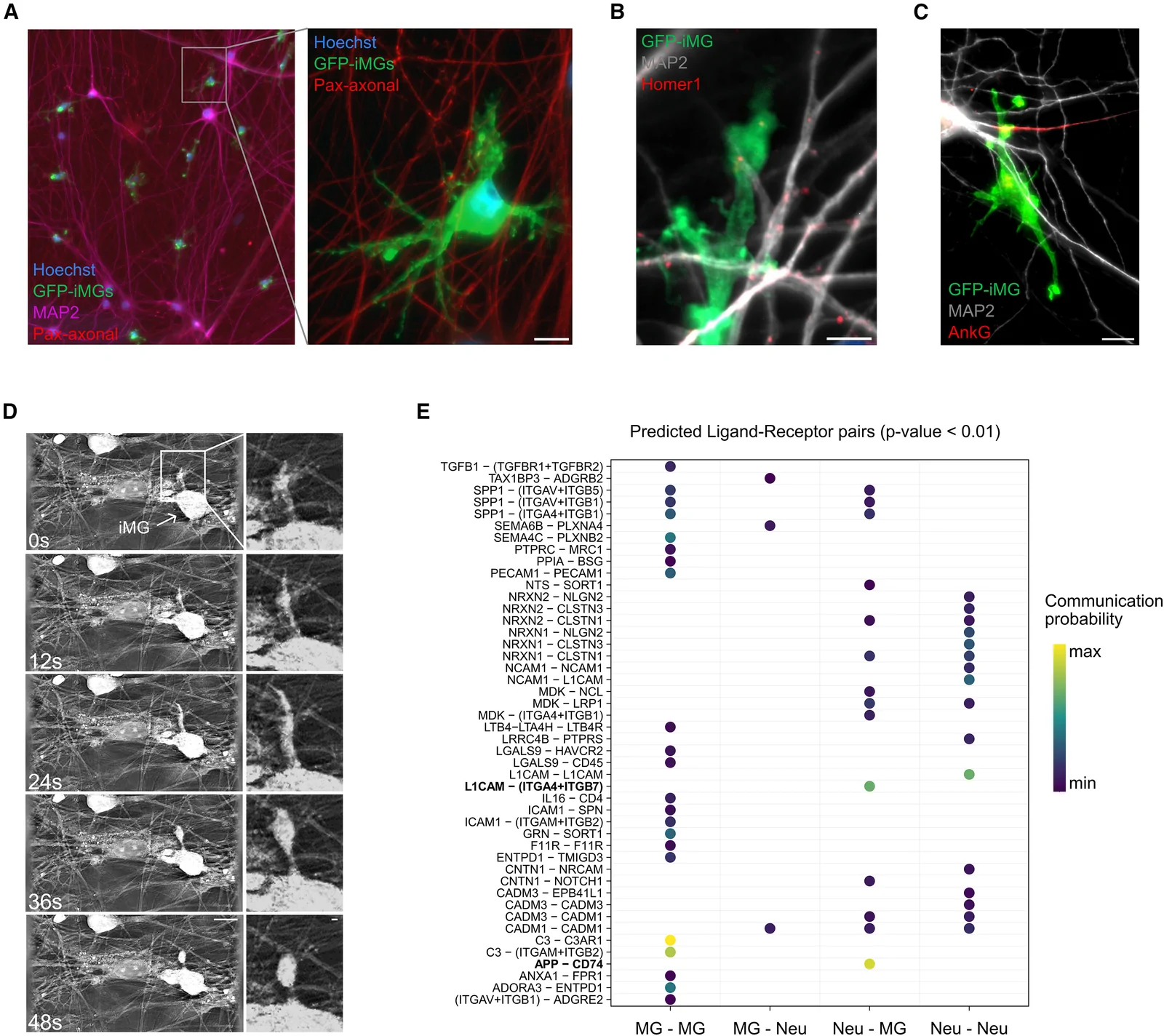
Co-maturation protocol allows for dynamic microglia-neuron interactions at dendrite, axon, and synapse level (A) Representative fluorescence microscope image of suggested microglial interaction with neuronal axons (pan-axonal) at DIV (days *in vitro*) 28, z stack shown as maximum projection (right); scale bar, 10 μm. (B) Representative fluorescence microscope images of microglial interaction with post-synapse (Homer1); scale bar, 5 μm (C) Representative fluorescence microscope images of microglial interaction with axon initial segment (AnkG) of a neuron , scale bar, 10 μm at DIV 28. (D) Refractive index live-cell imaging timeline showing microglial process interacting with neuron at DIV 28; scale bar, 10 μm; zoom-in scale bar, 1 μm. (E) Predicted ligand-receptor pairs of microglia and neurons in co-maturation cultures based on single-cell RNA-seq data at DIV 28; *N* = 3, *n* = 24,791 single cells. iMGs for data in this figure were generated from AICS-0016, iNeurons were generated from GM25256.

*In vivo* research suggests interaction of microglia with key sites such as synapses (Paolicelli et al., 2011; Eyo et al., 2021) or the axon initial segment (AIS) (Benusa and Lafrenaye 2020). We confirmed these characteristic neuron-microglia interactions in our *in vitro* co-maturation model using immunocytochemistry. The images suggest that iMGs form contact sites with axons, the postsynaptic marker Homer1, as well as the AIS (Figures 6A–6C). Live-cell imaging further showed continuous and rapid movement of microglial processes within the neuron-glia network and interaction with neuronal branches (Figure 6D and Video S1).

Video S1. Microglia-neuron interactions using refractive index live-cell imaging

Next, we used the CellChat algorithm (Jin et al., 2025) to explore receptor-ligand interactions between iMGs and iNeurons in our single-cell transcriptomic dataset of the co-maturation cultures (Figure 6E). We observed expected microglial communication pathways such as TGF-β or complement (C3) signaling. For crosstalk between neurons and iMGs, the top predicted interactions were *L1CAM-(ITGA4 + ITGB7)* and *APP*-*CD74* (Figure 6E). Interestingly, *ITGB7* was among the top upregulated genes in co-culture iMGs compared to monoculture iMGs (Figure 4C), and this upregulation might be driven by interaction with neuronal adhesion molecules. These findings indicate that the long-term duration of our co-maturation approach allows for dynamic and extensive neuron-microglia interactions.

### hiPSC-derived astrocytes are compatible with the long-term iMG-neuron co-maturation protocol

**Figure 7:**
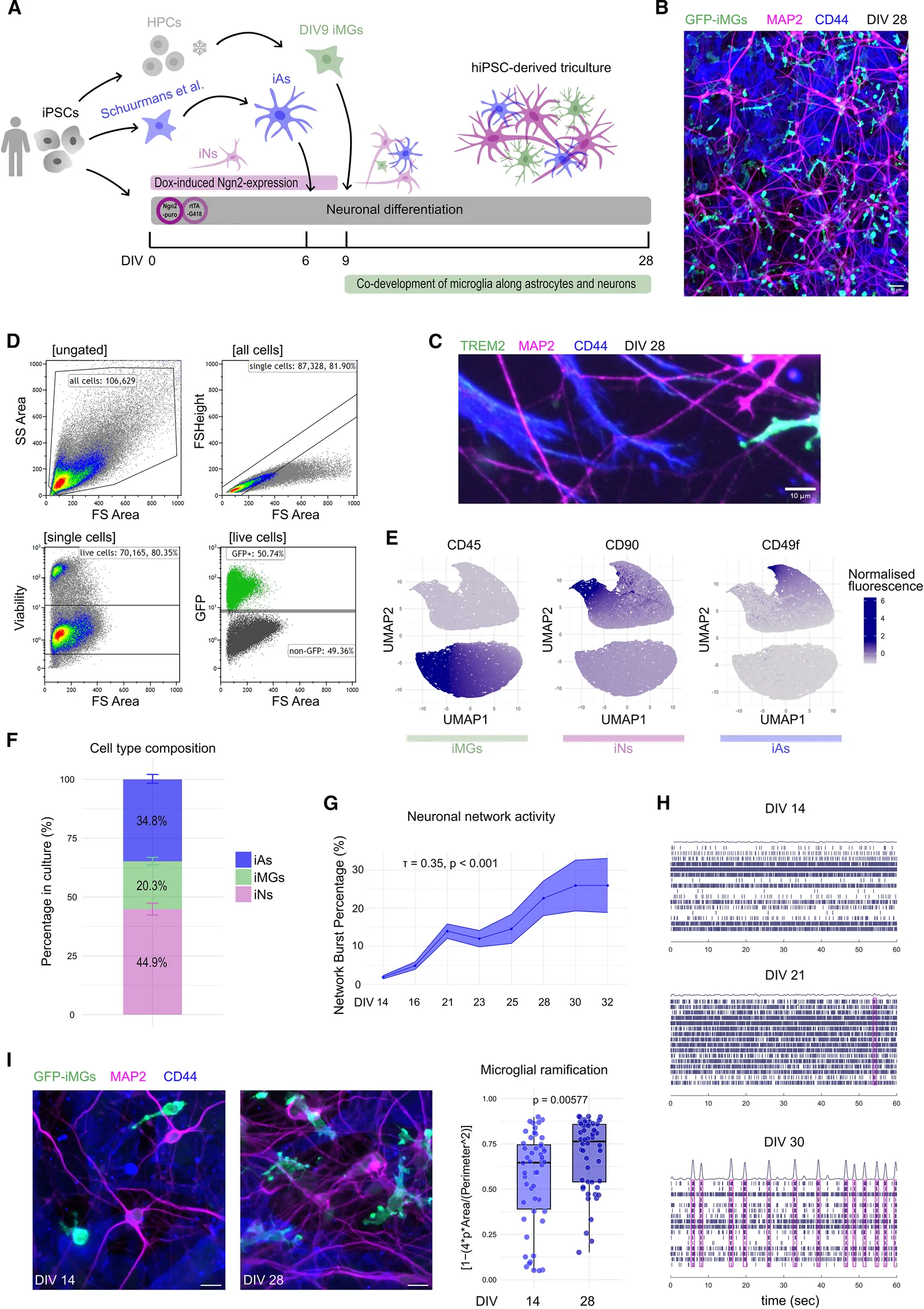
Human iPSC-derived astrocytes are compatible with iMG-neuron co-maturation protocol (A) Schematic protocol overview for generation of tricultures of hiPSC-derived astrocytes (iAs), microglia, and neurons. (B) Representative fluorescence microscope images showing iAs (CD44), iMGs (GFP), and neurons (MAP2) at DIV (days *in vitro*) 28; scale bar, 20 μm. (C) Representative fluorescence microscope images showing processes of iAs (CD44), iMGs (GFP), and neurons (MAP2) at DIV 28; scale bar, 10 μm. (D) Flow cytometry of hiPSC-derived tricultures showing gating strategy and presence of GFP-positive cells at DIV 28. (E) Uniform manifold approximation and projection (UMAP) analysis of CD45, CD90, and CD49f flow cytometry signal; *N* = 1, *n* = 70,000 single cells. (F) Cell-type composition in all human tricultures at DIV 28 quantified by immunocytochemistry images; *N* = 2, data represent means ± SEM. (G) Quantification of network burst percentage (%) across eight developmental time points; *N* = 1, *n* = 24 neuronal networks; data represent means ± SEM, Mann-Kendall test. (H) Representative micro-electrode array (MEA) raster plots with network bursts annotated in magenta showing increased organization of neuronal network activity from DIV14 to DIV 30. (I) Representative fluorescence microscope images of all human tricultures at DIV14 and DIV28; scale bar: 10 μm (left) and quantification of microglial ramification index across development, unpaired *t* test (right). iMGs for data in this figure were generated from AICS-0016; iNeurons and iAstrocytes were generated from GM25256.

To engineer a fully hiPSC-derived triculture, we successfully implemented our co-maturation protocol with hiPSC-derived astrocytes (iAs). The astrocyte part of the protocol is based on our previously optimized protocol (Schuurmans et al., 2026). As we developed those protocols alongside each other to achieve compatibility, they follow similar strategies. In short, hiPSCs were differentiated toward iAs for 42 days and added to *Ngn2*-induced neurons. The only modification to the rodent astrocyte co-maturation protocol was the introduction of human astrocytes at DIV 6 instead of DIV 2 (Figure 7A). The iMGs were added after doxycycline withdrawal at DIV 9, as previously done.

At DIV 28, we could still detect the GFP-tagged iMGs using both immunocytochemistry (Figures 7B and 7C) and flow cytometry (Figure 7D), confirming that the iAs were able to promote integration and long-term survival of iMGs. Furthermore, this showed that microglial survival was not driven by the *ex vivo* nature of the rodent astrocytes. Both iAs and iMGs contacted neuronal dendrites with their protrusions (Figure 7C). Using flow cytometry on the fully hiPSC-based tricultures, we reliably separated iMGs, neurons, and iAs with unsupervised clustering of surface-marker expression of CD45, CD90, and CD49f, confirming successful differentiation into all three respective cell types (Figure 7E).

Using immunocytochemistry, we assessed the cellular composition of fully hiPSC-derived tricultures at DIV 28 (Figure 7F) and found proportions comparable to those observed in co-maturation cultures with rodent astrocytes (Figure 3F). However, the proportion of iMGs was slightly higher (20% vs. ∼15%), suggesting that human astrocytes may provide greater support for microglial proliferation. Next, we assessed whether the fully hiPSC-derived triculture also supports synchronous network activity, a hallmark of high-quality hiPSC-derived neuronal networks as previously described (Mossink et al., 2021; Schuurmans et al., 2026). Indeed, we observed a progressive increase in network burst percentage over the course of development (Figures 7G and 7H), indicating the successful establishment of organized network activity. Microglia in the fully hiPSC-derived tricultures showed increasing ramification from DIV 14 to DIV 28 (Figure 7I), suggesting that the co-maturation protocol with neuronal networks using hiPSC-derived astrocytes also supports the development of microglial identity. Taken together, these findings highlight that our co-maturation protocol for iMGs and iNeurons can be successfully extended to astrocytes derived from hiPSCs.

## Discussion

Microglia are highly sensitive to their surroundings. This environment-dependent transcriptional and functional signature makes their *in vitro* modeling inherently challenging (Gosselin et al., 2017; Holtman et al., 2017). Consequently, monoculture systems of both primary and hiPSC-derived microglia are lacking essential CNS-derived signals required to recapitulate their physiological characteristics (Hanger et al., 2020; Jäntti et al. 2024; Schafer et al. 2023). Neurons and microglia continuously receive signals from each other influencing maturation and function. Thus, models used to understand neuron-microglia communication should reflect this ongoing interplay between microglia, neurons, and other CNS-resident cells such as astrocytes. To address this, we developed an hiPSC-based long-term culture model in which microglia and neurons co-mature. Our protocol offers a robust and efficient platform to comprehensively study human neuro-immune crosstalk.

The continuous interaction during co-maturation induced characteristic microglia-neuron interactions and improved the microglia signature on morphology, gene, and protein level. The presence of the hiPSC-derived neuron-glia networks led to pronounced microglial ramification similar to how microglia are observed in xenotransplanted organoids (Schafer et al., 2023). This characteristic microglial morphology is accompanied by an upregulation of markers associated with neuronal remodeling and homeostatic microglia (Paolicelli et al., 2022; Holtman et al., 2017), highlighting the importance of contact with other CNS-resident cells for establishing microglia identity *in vitro*. Interestingly, genes and signaling pathways associated with neurodegenerative pathology were strongly downregulated in microglia in the co-cultures compared to monocultures. These included *APOE* and *SPP1*, which have been widely studied for their role in AD (Gosselin et al., 2017; Lan et al., 2024; Sun et al., 2023; Li et al., 2025; van Olst et al., 2025). Our findings may suggest that a loss of healthy signaling from neurons might drive the characteristic disease-associated phenotype both in microglia from AD patients and in microglia monocultures. This further highlights that our co-maturation model could mitigate the stress typically induced by culturing microglia *in vitro*, which normally skews them toward a neurodegenerative-associated substate. As a result, our co-culture provides a suitable platform for studying microglial roles in a variety of contexts.

During optimization of our co-maturation model, we discovered a fundamental role of astrocytes for microglial survival and integration. Several studies have highlighted microglia-astrocyte crosstalk in the past (Damisah et al., 2020). Especially the astrocytic secretion of growth factors has been shown to be critical for microglia differentiation (Butovsky et al., 2014). However, it is likely that the supportive function of astrocytes extends beyond the supply of trophic factors such as TGF-β and M-CSF (Erblich et al., 2011), as we supplemented both monocultures and co-cultures with these factors (see STAR Methods). Moreover, the protective effect of astrocytes might also be mediated indirectly via effects on the neurons. The survival of microglia in monoculture, but not in neuron-only co-cultures, suggests that neurons may release stress factors when astrocytes are absent. Astrocytes promote neuronal growth (Banker 1980) and synaptic maturation (Zhang et al., 2013) in cultured neurons and are therefore widely used for *in vitro* studies of neuronal function (Frega et al., 2017). While neuronal numbers remained stable when cultured without astrocytes, their health and maturity may have been compromised. This could in turn limit microglial health and integration into the neuronal networks. Next to rodent astrocytes, we also streamlined this co-maturation protocol for use with our hiPSC-derived astrocyte protocol (Schuurmans et al., 2026). This allows for the fast and robust generation of a fully hiPSC-derived triculture with neurons, astrocytes, and microglia, expanding the possibilities in studying human brain function.

Advances in hiPSC technologies have yielded complex 3D models (Schafer et al., 2023; Stanton et al., 2025) such as organoids for *in vitro* studies. While these models have advantages depending on the research question, especially for understanding cortical development, they are challenging to scale up for high-throughput screening. The 2D format enhances scalability and facilitates applications such as (live-cell) imaging, MEAs, and drug screening. This protocol allows for using cost-effective high-throughput pipelines while maintaining sufficient complexity to model cell-cell interactions and functional phenotypes that reflect physiological conditions. It is therefore particularly well suited to disentangle the role of microglia in neuronal development and function (Mordelt et al., 2026).

Furthermore, our co-maturation approach allows for full control of the genotype of microglia, neurons, and astrocytes, making it especially useful to disentangle cell-type-specific contributions to disorders with a known genetic component. We provide troubleshooting tips for our co-maturation approach (e.g., working with disease lines, detachment issues, remaining hiPSC colonies, clustering) in Table S1. Variations of the co-maturation protocol to accommodate specific requirements can be made (e.g., human or rodent astrocytes, addition of inhibitory neurons; Wang et al., 2023). Our improved co-maturation model of hiPSC-derived microglia with neuron-glia networks provides a powerful tool to investigate neuro-immune crosstalk in health and disease and specifically elucidate the role of microglia in modulating neuronal function and development.

## Resource availability

### Lead contact

Requests for further information and resources should be directed to and will be fulfilled by the lead contact, Lot de Witte (lot.dewitte@radboudumc.nl).

### Materials availability

This study did not generate new unique reagents.

### Data and code availability

• Source data of transcriptomic and MEA analyses underlying Figures 2, 4, and 5 are available in Table S2.
• The raw single-cell RNA-seq data are deposited in the GEO repository (GSE339528).
• All other raw datasets generated during the current study are available from the lead author on reasonable request.

## Acknowledgments

We thank all members of the de Witte and Nadif Kasri labs as well as Marijn Kuijpers for their valuable input during meetings and discussions. We thank Jorge Domínguez Andrés for kindly contributing the human monocyte samples. We also thank the RadboudUMC Flow Core for supporting experiments as well as providing broncho-alveolar fluids. This work was supported by the Hypatia Grant from the RadboudUMC, the 10.13039/100000893Simons Foundation (00010410), and the Netherlands organization for health research (10.13039/501100001826ZonMW Open Competitie 09120012110034).

## Author contributions

A.M., N.N.K., and L.D.d.W. conceptualized the study. N.N.K., D.S., and L.D.d.W. supervised the study. A.M. wrote the original draft of this manuscript and was responsible for the visualization of the data. I.M.E.S., N.S., M.P.H., N.N.K., D.S., and L.D.d.W. reviewed and edited the manuscript. A.M. developed and optimized the co-maturation protocol. I.M.E.S. assisted with conceptualization and execution of the astrocyte integration. K.S. performed RNA isolations. K.M. performed and analyzed microglial ramification. M.G. performed phagocytosis live-cell imaging and provided validation in additional lines. C.O.G. performed culturing for secretome assessment. K.W.M. and L.J.A.W. conceptualized and reviewed the single-cell transcriptomics experiments. L.J.A.W. and A.M. performed and analyzed the single-cell transcriptomics experiments. A.M. performed and analyzed the multi-electrode array assays. N.S. and M.P.H. performed the single-cell electrophysiology experiments. A.M. performed the overall data analysis and integration across experimental readouts.

## Declaration of interests

The authors declare no competing interests.

## STAR★Methods

### Key resources table

**Table:** 

REAGENT or RESOURCE	SOURCE	IDENTIFIER
**Antibodies**		

Anti-CX3CR1, rabbit polyclonal	Invitrogen	Cat#PA5-19910; RRID:AB_2538394
Anti-TREM2, rat monoclonal	R&D Systems	Cat#MAB17291-SP
Anti-P2RY12, rabbit polyclonal	Alomone Labs	Cat#APR-012; RRID:AB_2337543
Anti-TMEM119, mouse monoclonal	BioLegend	Cat#A16075D
Anti-MAP2, guinea pig polyclonal	Synaptic Systems	Cat#188004; RRID:AB_2147440
Anti-panAxonal (SMI-312), mouse monoclonal	BioLegend	Cat#837904; RRID:AB_2566782
Anti-GFAP, rabbit polyclonal	Sigma-Aldrich	Cat#AB5804; RRID:AB_2109645
Anti-Synapsin I, rabbit polyclonal	Sigma-Aldrich	Cat#AB1543P; RRID:AB_2200400
Anti-Homer1b/c, mouse monoclonal	Synaptic Systems	Cat#160111; RRID:AB_887730
Anti-Ankyrin-G, mouse monoclonal	Invitrogen	Cat#33-800; RRID:AB_2533145
CD43-PE (clone 10G7)	BioLegend	Cat#343203; RRID:AB_1877299
CD45-FITC	eBioscience	Cat#11-9459-42
CD45-PE/Cyanine7	BioLegend	Cat#304015; RRID:AB_493729
CD11b-PE	BioLegend	Cat#301305; RRID:AB_314152
CX3CR1-APC	BioLegend	Cat#341110; RRID:AB_11125577
HLADR-APC	eBioscience	Cat#47-9956-42
P2RY12-BV421	BioLegend	Cat#392105; RRID:AB_2750213
CD206-APC	BD Biosciences	Cat#550889; RRID:AB_393992
CD24-PE Cyanine7	BioLegend	Cat#311119; RRID:AB_2561736
GFAP-BV421	BioLegend	Cat#644710; RRID:AB_2734370
Secondary antibodies, Alexa Fluor 488/568/647	Invitrogen	1:1000

**Bacterial and virus strains**		

Lentiviral ChR2-eYFP (human Synapsin I promoter)	Addgene	Cat#20945; RRID:Addgene_20945
Lentiviral Ngn2 overexpression construct	Addgene	Cat#198754 RRID:Addgene_198754
Lentiviral rtTA construct	Addgene	Cat#198756 RRID: Addgene_198756

**Biological samples**		

Human post-mortem brain tissue-derived microglia	Netherlands Brain Bank	N/A
Alveolar monocytes (bronchoalveolar lavage fluid)	Radboudumc	N/A

**Chemicals, peptides, and recombinant proteins**		

Essential 8™ Flex Basal Medium	Gibco	Cat#A2858501
Primocin	Invivogen	Cat#ANT-PM-2
Geltrex reduced growth factor basement membrane matrix	Gibco	Cat#A1413301; Cat#A1413202
ReLeSR	Stemcell Technologies	Cat#100-0483
PSC Cryomedium	Gibco	Cat#A2644601
DMEM/F12	Gibco	Cat#11320074
GlutaMAX Supplement	ThermoFisher	Cat#35050061
B-27 Supplement (50x)	Gibco	Cat#17504044
IL-34	Peprotech	Cat#200-34
TGF-β1	Peprotech	Cat#167100-21
M-CSF	Peprotech	Cat#167300-25
GM-CSF	Peprotech	Cat#300-03
TrypLE Express	Gibco	Cat#12604021
Poly-L-ornithine (PLO)	Sigma	Cat#P3655
Human Laminin LN521	BioLamina	Cat#LN521-05
E8 Basal Medium	Gibco	Cat#A1517001
RevitaCell™ Supplement (100x)	Gibco	Cat#A2644501
Doxycycline hyclate	Sigma	Cat#D5207
G418 (Geneticin)	Sigma-Aldrich	Cat#G8168
Puromycin dihydrochloride	Sigma	Cat#P9620
N-2 Supplement (100x)	Gibco	Cat#17502001
NT-3	Peprotech	Cat#450-03
BDNF	Peprotech	Cat#450-02
MEM Non-Essential Amino Acids Solution	Sigma-Aldrich	Cat#M7145
Cytosine β-D arabinofuranoside (Ara-C)	Sigma-Aldrich	Cat#C1768
Neurobasal Medium	Gibco	Cat#21103049
Fetal bovine serum (FBS)	Sigma	Cat#F7524
Hydrocortisone	Sigma-Aldrich	Cat#H0888; CAS:50-23-7
ATP	Sigma-Aldrich	CAS:74804-12-9
TNFα	Peprotech	Cat#300-01A
IFNγ	Peprotech	Cat#300-02
Triton X-100	Sigma-Aldrich	Cat#T8787
Bovine serum albumin (BSA)	Sigma	Cat#A7906
Hoechst 33342	ThermoFisher	Cat#H3570
DAKO Mounting Medium	Agilent	Cat#S3023
Viability Dye eFluor 780	Invitrogen	Cat#65-0865-14
Human TruStain FcX Blocking Solution	BioLegend	Cat#422302
DNA/RNA Shield	Zymo Research	Cat#ZY-R1200-125
Tetrodotoxin (TTX)	Tocris	Cat#1069
Picrotoxin	Tocris	Cat#1128
NBQX disodium salt	Tocris	Cat#0373
Accutase	Sigma-Aldrich	Cat#A6964

**Critical commercial assays**		

HPC Kit	Stemcell Technologies	Cat#05310
MycoAlert PLUS Mycoplasma Detection Kit	Lonza	Cat#LT07-703
Chromium Next GEM Single Cell Fixed RNA Sample Prep Kit	10x Genomics	Cat#PN-1000414
Chromium Single Cell Flex Kit	10x Genomics	Protocol CG000527 Rev F
Quick-RNA Microprep Kit	Zymo Research	Cat#ZY-R1051
NEBNext® Ultra™ II RNA Library Prep Kit	NEB / Illumina	N/A
Compensation beads	BioLegend	Cat#424602

**Deposited data**		

Single-cell RNA sequencing data for iMGs in monoculture or iMGs in neuronal network co-culture	GEO repository	GEO:GSE339528

**Experimental models: Cell lines**		

Human: WTC-11 hiPSC	Coriell Institute	Cat#GM25256; RRID:CVCL_Y803
Human: WTC-11 endogenous Actin-GFP hiPSC	Allen Institute for Cell Science	Cat#AICS-0016 cl.184
Human: HPSI0314i-hoik_1 hiPSC	HipSci / Wellcome Trust Sanger Institute	RRID:CVCL_TBD
Human: HPSI0114i-kolf_2 hiPSC	HipSci / Wellcome Trust Sanger Institute	RRID:CVCL_TBD

**Experimental models: Organisms/strains**		

Rat: Wistar, embryonic day 18 (E18) cortical astrocytes	Radboud University / Frega et al.^30^	N/A

**Software and algorithms**		

R v4.2.0 / RStudio	R Core Team	https://www.r-project.org/; RRID:SCR_001905
GraphPad Prism v8.0.0	GraphPad Software	https://www.graphpad.com/; RRID:SCR_002798
pheatmap R package	CRAN	https://CRAN.R-project.org/package=pheatmap
Seurat v4.3.0	Satja lab	https://satijalab.org/seurat/; RRID:SCR_007322
Harmony v1.2.3	Korsunsky et al. (2019)	https://github.com/immunogenomics/harmony; RRID:SCR_023206
CellChat v2.1.2	Jin et al. (2025)	https://github.com/jinworks/CellChat
Cell Ranger v9.0.0	10x Genomics	https://www.10xgenomics.com/support/software/cell-ranger; RRID:SCR_023221
nf-core/rnaseq pipeline v3.18	nf-core	https://github.com/nf-core/rnaseq
STAR aligner	Alex Dobin	https://github.com/alexdobin/STAR; RRID:SCR_004463
Salmon	Combine lab	https://combine-lab.github.io/salmon/; RRID:SCR_017036
FastQC	s-andrews	RRID:SCR_014583; https://github.com/s-andrews/FastQC
MultiQC	Scilifelab	RRID:SCR_014982
Trim Galore	Felix Krueger	RRID:SCR_011847; https://github.com/FelixKrueger/TrimGalore
Kaluza C Analysis Software	Beckman Coulter	RRID:SCR_016182
AxIS Navigator	Axion Biosystems	N/A
NeuralMetric Tool	Axion Biosystems	N/A

**Other**		

CytoView 48-well MEA plates	Axion Biosystems	Cat#M768-tMEA-48W
Axion Maestro Pro MEA recording system	Axion Biosystems	N/A
Zeiss Axio Imager Z2	Zeiss	N/A
Gallios flow cytometer	Beckman Coulter	N/A
Multiclamp 700B amplifier	Molecular Devices	N/A
Digidata 1440A digitizer	Molecular Devices	N/A
KSL2 470 nm LED light source	Rapp OptoElectronic	N/A
PC-10 micropipette puller	Narishige	N/A
K-7400 Semi-Micro Osmometer	Knauer	N/A
NovaSeq X+ sequencer	Illumina	N/A
Mr. Frosty Freezing Container	Thermo Fisher	N/A
6-well plates	Corning	Cat#353046

### Experimental model and study participant details

#### Human induced pluripotent stem cell lines and maintenance

Commercially available and quality controlled human induced pluripotent stem cell lines (hiPSCs) were used. These include WTC-11 (Coriell Institute, GM25256), and WTC-11 with an endogenous actin-GFP tag (Allen Institute, AICS-0016 cl.184), HPSI0314i-hoik_1, and HPSI0114i-kolf_2 (Human Induced Pluripotent Stem Cells Initiative, Wellcome Trust Sanger Institute). All hiPSC lines are fibroblast derived. WTC-11 underwent episomal reprogramming, and the HPSI lines were reprogrammed with non-integrating Sendai virus. All available metadata and the full characterization (passage number, pluripotency testing, etc.) can be found on the respective vendor website. In addition to the karyotyping performed by the vendor, we genetically profiled the hiPSC lines using droplet digital PCR before cryopreserving. HiPSCs were cultured in Essential 8™ Flex Basal Medium (Gibco, A2858501) supplemented with Primocin (0.1 μg/ml, Invivogen; ANT-PM-2) on Geltrex-coated (Gibco, A1413301) 6-well plates (Corning, 353046) at 37°C/5% CO2. Medium was refreshed every 2-3 days. At around 80% confluency the hiPSCs were passaged (splitting ratio 1:10 to 1:30) using the enzyme-free reagent ReLeSR (Stemcell Technologies, 100-0483). Passaging was done with wide-tip pipettes to keep hiPSC colonies intact and reduce stress. Experiments were generally performed not later than 10-20 passages of the purchased original vial. All hiPSCs were tested biweekly for mycoplasma contamination using MycoAlert PLUS (Lonza, LT07-703), and routinely checked for bacterial or fungal infections during cell handling. HiPSCs were frozen in a Mr. Frosty Freezing Container (Thermo Fisher) using PSC Cryomedium (Gibco, A2644601) and stored in liquid nitrogen. All differentiations were initiated only after the hiPSCs had been cultured for at least two weeks following vial thawing.

### Method details

#### iMicroglia differentiation

The differentiation of hiPSC-derived microglia (iMGs) follows a two-step protocol with initial hematopoietic progenitor cell (HPC) generation (Figure S2A), an optional freezing step for streamlining, and subsequent microglia differentiation. HPCs were generated with the HPC Kit from Stemcell Technologies according to the guidelines provided if not stated otherwise (05310). Assessing marker expression over the timeline of differentiation revealed that HPCs are best harvested at DIV 11 (Figure S2). We restrained for multiple DIV harvest to ensure overall quality and reduce variability in differentiations. During our optimization we found that HPC quality determines the success of the microglia differentiation, and we therefore only used harvests that showed >95% of CD43 and CD45 positive cells. The HPCs were frozen in a Mr. Frosty Freezing Container (Thermo Fisher) using PSC Cryomedium (Gibco, A2644601) and stored in liquid nitrogen at 500k/vial until further use.

HPCs were thawed in neuron-compatible microglia medium. This medium consists of DMEM/F12 base (Gibco, 11320074) supplemented with GlutaMAX (1x, ThermoFisher, 35050061), B27 (1x, Gibco, 17504044), Primocin (100 μg/ml, Invivogen, ant-pm-2), IL34 (100 ng/ml, Peprotech, 200-34), TGF-b (50 ng/ml, Peprotech, 167100-21), M-CSF (25 ng/ml, Peprotech, 167300-25). To maximize cell survival, thawing must be conducted fast. Standard pipette tips can be used due to the single-cell nature of HPCs. HPCs were seeded onto six-well plates coated with Geltrex (1:100, Gibco, A1413202) at a density of 250,000 HPCs per well. We found that a lower starting density is not beneficial for microglia differentiation. Throughout the differentiation process, iMG cultures were refreshed 2-3 times a week by adding one milliliter per well in a six-well plate without media aspiration to retain floating microglia. After around 5 days, the wells became highly confluent and the iMGs were split by transferring the floating iMGs to new TC-treated wells that do not require additional coating for microglial attachment after the first 3-5 days. In this first split, we supported proliferation and survival by addition of GM-CSF (10 ng/ml, Peprotech, 300-03). However, during the following splits and the subsequent culture process, we restrained using it to not compromise on microglia phenotype.

During culturing the next weeks, microglial cultures were split upon the observation of cell aggregates or medium overflowing. This typically occurred around DIV 8 and DIV 15 but depended on the cell line and batch used. Splitting strategy was optimized to maximize cell health under different circumstances. If no extreme cell aggregates were observed (e.g. the first splitting was primarily performed to prevent medium overflowing) only the floating cells were transferred to new wells, avoiding the detachment of adherent iMGs. This approach minimizes centrifugation stress, preserves ramified microglial morphology and improved differentiation outcomes. In case GM-CSF was used (first split) it was also added in equal concentration to the original well. In case the iMGs formed clumps that continued to grow, cells were dissociated with TripLE (Gibco, 12604021) using a P1000 pipette. The collected cells were centrifuged at 400 × g for 5 min, resuspended in fresh base medium with cytokines, and replated in new TC-treated wells at appropriate densities. With both splitting methods, the transferred cells attached and showed no differences in morphology or marker expression compared to the original adherent cells from the starting wells.

We observed that microglial proliferation was high in the initial ten days but declined over time. Therefore, we restrained for splitting late during the differentiation process. Low density of iMGs (>250k per well in 6-well plate) limited cell survival. Example pictures of iMG cultures and recommendations on when to split can be found in Figure S7. Throughout the differentiation period, cultures were monitored for morphology, and appropriate density adjustments were made to optimize iMG yield and phenotype.

#### *Ngn2*-induced neuronal differentiation

We differentiated hiPSCs to excitatory cortical neurons as previously described (Frega et al., 2017), but introduced minor adjustments to allow for microglia co-culture. HiPSCs were kept at 37°C/5%/CO2. To allow differentiation, hiPSCs were transduced with lentiviral *Ngn2* and rtTA constructs and selected with increasing concentrations of G418 (100 - 250 μg/ml, Sigma-Aldrich, G8168) and puromycin (1 - 2 μg/ml, Sigma; P9620) respectively. Upon successful lentiviral integration, neuronal differentiation by *Ngn2* overexpression can be initiated after seeding single cells by doxycycline treatment.

Coverslips for immunocytochemistry were treated with nitric acid for three days, washed 20 times with ultra-pure water and sterilized at 180°C for 5 h to ensure a clean surface for attachments of the neuron. To support attachment of the neurons, the wells or coverslips were first coated with poly-L-ornithine (50 μg/ml, PLO, Sigma, 3655) diluted in borate buffer for at least 3 h. After washing with ultra-pure water without drying out the wells, the wells were incubated with human laminin (5 μg/ml, Biolamina, LN521-05) overnight at 4°C. Induction of neuronal differentiation (DIV 0) was done by dissociating hiPSC colonies into single cells using TrypLE and seeding onto the pre-coated wells in E8 basal medium (Gibco; A1517001) supplemented with RevitaCell™ (1:100, Gibco, A2644501), doxycycline (4 μg/ml, Sigma, D5207), and Primocin (100 μg/ml, Invivogen, ant-pm-2). On MEA-chips, droplet-plating was performed to ensure successful coverage of the electrodes. We plated 35k cells in 24-well plates with coverslips for immunocytochemistry, 300k cells in 6-well plates for flow cytometry and 26k cells on Cytoview 48-well MEA plates (Axion Biosystems, M768-tMEA-48W) for neuronal activity recordings. The day after plating (DIV 1) a full medium change to DMEM/F12 (Gibco, 11320074) supplemented with 1x N2 (Gibco, 17502001), 10 ng/mL NT3 (Peprotech; 450-03), 10 ng/mL BDNF (Peprotech; 450-02), 100 μM MEM non-essential amino acid solution (NEAA; Sigma-Aldrich; M7145), 4 μg/μl doxycycline, and 100 μg/ml Primocin was performed.

Neuronal maturation was supported by embryonic (E18) rat astrocytes, which were added to the culture in a 1:2 ratio at DIV 2. The procedure of isolation and culturing of these rat astrocytes is described in detail in Frega et al., (2017). For the addition of hiPSC-derived astrocytes to *Ngn2*-induced neurons in a 1:2 ratio at DIV 6, please see our optimized protocol(Schuurmans et al., 2026). The neuronal culture component is identical to this one as both protocols are streamlined to allow for hiPSC-derived tricultures of neurons, astrocytes, and microglia. The manuscript provides a step-by-step guide to astrocyte differentiation including practical recommendations.

From DIV 3 onwards, the medium was switched to neurobasal medium (Gibco, 21103049) supplemented with B-27, GlutaMAX, Primocin, NT3, BDNF, and doxycycline. To remove proliferating hiPSC colonies and astrocytes from the cultures Cytosine β-D arabinofuranoside (Ara-C; 1 μM; Sigma-Aldrich; C1768) treatment was performed once at DIV 3. From DIV 6 onwards, a half medium change was performed three times a week (Mon, Wed, Fri). To prevent stress on the neurons and circumvent artifacts for later microglia addition, doxycycline was withdrawn from the medium starting at DIV 8 in all conditions. FBS was added to support astrocytic survival from DIV 9 onwards (2.5 % Sigma, F7524). Wells with unequal cell densities or uneven distribution (clustering, highway-formation, see troubleshooting tips) were excluded from analysis. The cultures were kept at 37°C/5% CO2.

#### Microglia-neuron co-maturation culture

Microglial and neuronal differentiation were initiated according to the guidelines described above. The microglial culture was always started on day prior to the neuronal differentiation. At DIV 9 of the neuronal differentiation corresponding to DIV 10 of microglial differentiation, they were combined. First, a half-medium change was performed to introduce the three microglial-supporting cytokines IL34, TGFβ, and M-CSF to the neuronal cultures. To avoid potential effects on the differentiating microglia, it is important that doxycycline was subsequently omitted from the medium. The complete composition of the co-maturation medium is Neurobasal medium (Gibco, 21103049) supplemented with GlutaMax (1x, ThermoFisher, 35050061), B27 (1x, Gibco, 17504044), Primocin (100 μg/ml, Invivogen, ant-pm-2), NT3 (10 ng/ml, Preprotech, 450-03), BDNF (10 ng/ml, Peprotech, 450-02), FBS (2.5%, Sigma, F7524), IL34 (100 ng/ml, Peprotech, 200-34), TGFβ (50 ng/ml, Peprotech, 167100-21), M-CSF (25 ng/ml, Peprotech, 167300-25). We found that withdrawal of FBS and consequently the formation of reactive astrocytes (based on their morphology) is more detrimental for microglial activation and survival than the addition of FBS. Therefore, we comprised with adding FBS at low dosage of 2.5%.

To not disrupt the cultures and remove secreted growth factors, it is important that the medium transition was conducted as a half-medium change. We therefore removed half the medium from the wells with the neurons, prepared the co-maturation medium with double the concentration of the cytokines IL34, TGFβ, and M-CSF and added it in a 1:1 ratio to the existing medium. This is possible because the compositions of the neuronal and co-maturation medium are identical, except for the inclusion of three cytokines. Then, the plates were incubated at 37°C/5% CO_2_ for at least 30 min to calibrate temperature and CO_2_ levels before adding iMGs. The iMGs were harvested with TrypLE (Gibco, 120604021), centrifuged (400g) at room temperature for 5 min and resuspended in co-maturation medium to obtain a single-cell suspension. After counting with Trypan blue, iMGs were added to the neurons in a 1:5 ratio. In 24-well we plated 35k neurons, 20k astrocytes, and 7k iMGs. In 6-well plates, we plated 150k neurons, 100k astrocytes, and 30k microglia. In 48-well MEA plates (Axion Biosystems) we plated 26k neurons, 13k astrocytes, and 5.2k microglia.

The iMGs can be added into the full medium (no droplet plating needed) as they move towards the neuron-glia networks and integrate by themselves. Half of the medium was refreshed with co-maturation medium three times a week (Mon, Wed, Fri). Medium was always carefully taken from the upper edge of the well to not disrupt microglia integration into the cultures. The co-cultures were kept at 37°C/5% CO_2_ throughout the entire differentiation process.

#### Pharmacology

All reagents were prepared into concentrated stocks and stored at −20°C. For stimulations of microglia cultures, we used hydrocortisone (2.5 μM, Sigma-Aldrich, H0888), ATP (1 mM, Sigma-Aldrich, 74804-12-9), TNFα (100 ng/ml, Peprotech, 300-01A) and IFNy (100 ng/ml, Peprotech, 300-02). All concentrations were tested for potential toxicity effects and cell death before. If possible, compounds were diluted in ultra-pure water (TNFα, IFNy, ATP). Otherwise, dimethyl sulfoxide (DMSO) was used (hydrocortisone). For all pharmacological treatments and experiments, the amount of DMSO in the cell culture medium was ≤0.5% v/v.

#### Immunocytochemistry

Unless stated otherwise, subsequent steps were performed at room temperature and solutions were made in 1XPBS. Cultured cells were fixed by using a gradient of 2% and subsequently 4% paraformaldehyde (PFA) supplemented with 4% sucrose. First, PFA was added to the culture in a 1:1 ratio with existing medium. After 7 min, all the medium was carefully removed from the well and 4% PFA was added for additional 7 min. We found that this process preserved cellular morphologies compared to a direct change to PFA from medium. After PFA incubation, cells were washed three times with 1XPBS, and permeabilized with 0.2% Triton (Sigma-Aldrich, T8787) for 10 min. To block nonspecific binding sites, cells were incubated with 3% bovine serum albumin (BSA, Sigma, A7906) for 1 h. Primary antibodies were diluted in the same buffer and incubated overnight at 4°C. The following primary antibodies and dilutions were used: rabbit anti-CX3CR1 (1:300, Invitrogen, PA5-19910), rat anti-TREM2 (1:300, R&D Systems, MAB17291-SP), rabbit anti-P2RY12 (1:300, Alomone Labs, APR-012), mouse anti-TMEM119 (1:300, Biolegend, A16075D), guinea pig anti-MAP2 (1:1000, Synaptic Systems, 188004), mouse anti-panAxonal (1:1000, BioLegend, 837904), rabbit anti-GFAP (1:1000; Sigma-Aldrich, AB5804), rabbit anti-Synapsin I (1:500, Sigma-Aldrich, AB1543P), mouse anti-Homer1b/c (1:200, Synaptic Systems, 160111), mouse anti-ankyrin-G (1:1000, Invitrogen, 33-800).

Secondary antibodies conjugated to either Alexa Fluor 488, 568, or 647 (Invitrogen) were incubated for 1h at 1:1000 in 1% BSA. Cell nuclei were stained with Hoechst (0.01%, ThermoFisher, H3570) for 10 min. After three final washes, the coverslips were mounted in DAKO (Agilent, S3023) on microscope slides and imaged on a Zeiss Axio Imager Z2. For microglial survival and cell type composition and analysis 20x magnification was used. For morphological and fluorescence analysis per cell x63 magnification with oil was used.

#### Flow cytometry

Cultured cells were washed with 1xPBS and dissociated into a single-cell suspension using TrypLE (Gibco, 120604021). The cells were dissolved in Viability Dye eFluor 780 (Invitrogen, 65-0865-14,) and incubated at 4°C for 30 min. Subsequently, cells were centrifugated at 300xg for 5 min and washed with flow buffer (1% BSA in PBS) twice. Then, cells were fixed for 15 min at RT with 2% paraformaldehyde followed by another wash. To prevent aspecific binding to Fc-receptors, the cells were blocked with 2.5% Human TruStain FcX blocking solution (BioLegend, 422302) for 10 min. Following antibodies were used: CD43-PE (1:100, BioLegend, 343203), CD45-FITC (1:200, eBioscience, 11-9459-42), CD45-PE/Cyanine7 (1:200, BioLegend, 304015), CD11b-PE (1:100, BioLegend, 301305), CX3CR1-APC (1:100, BioLegend, 34110), HLADR-APC (1:50, eBioscience, 47-9956-42), P2RY12-BV421 (1:100, BioLegend, 392105), CD206-APC (1:50, BD Biosciences, 550889), CD24-PE Cyanine7 (1:50, BioLegend, 311119), GFAP-BV421 (1:100, BioLegend, 644710).

After 30 min incubation at 4°C, the cells were washed three times and finally dissolved in 200 μl flow buffer for flow cytometry measurement (Gallios, Beckman Coulter). Gating of side scatter (SS) area versus forward scatter (FS) area was used to define the cell population. Duplicates were removed by gating between FS height and FS Area. Further, dead cells were negatively gated by viability dye intensity. Unstained samples were used to determine the auto fluorescence levels of cells in the channels corresponding to the antibodies used. The compensation strategy was determined using beads (BioLegend, 424602). Analysis was performed using Kaluza C Analysis software (Beckman Coulter).

#### RNA-sequencing

Plating and culturing procedure was done according to the specific cell type. HiPSCs, iMGs, and iNeurons were cultured as described in this manuscript. Alveolar monocytes were isolated from residual biomaterial from broncho-alveolar fluid from patients visiting Radboudumc that did not show pathology phenotypes and had provided informed consent for the use of leftover biological material for scientific research purposes. Macrophages were derived from blood-isolated monocytes. The primary human post-mortem tissue derived microglia were obtained from the Netherlands brain bank and stored in Trizol. For detailed information on the isolation and maintenance of primary microglia from human post-mortem brain tissue, please see our previous work(Lopes et al., 2022). Cultured cells were washed with 1xPBS and collected using DNA/RNA shield (Zymo Research; ZY-R1200-125).

RNA was isolated using the Quick-RNA Microprep kit (Zymo Research, ZY-R1051) according to manufacturer’s instructions. After quality control, NEBNext® Ultra™ II RNA Library Prep Kit (Illumina) was used for library preparation as per manufacturer`s recommendations. Sequencing was performed on a NovaSeq X+ (Illumina) at 2x150bp configuration with coverage of 20 million reads per sample. Raw RNA-sequencing reads were processed using the nf-core/rnaseq pipeline (v3.18) implemented in Nextflow. Quality control was performed using FastQC and MultiQC. Adapter trimming was conducted with Trim Galore. Reads were aligned to the human reference genome (GRCh38) using STAR with default parameters. Transcript-levels were quantified with Salmon. Log2 transformation with a pseudo-count of +1 was performed on counts per million (CPM) to achieve normal distribution. Key microglial marker genes were assessed in the iMG samples compared to other cell types and across development. SALL1 was excluded from the analysis because it was highly expressed in the starting iPSCs, consistent with its dual role in both embryonic development and microglial identity. Heatmaps were generated using Euclidean clustering in the *pheatmap* R package.

#### Single-cell RNA-sequencing

Microglia monocultures and neuron-microglia co-cultures were differentiated in 6-well plates according to their protocol described above. The microglia monocultures also received PLO/human laminin coating as the co-cultures for fair comparison. The experiment was performed in technical triplicates per condition. At DIV 28 the cultures were dissociated using Accutase (20 min at 37°C). All conditions were treated exactly the same to ensure a fair comparison, even though microglia monocultures did not require as long an incubation period as the very dense neuron-glia networks. The single-cell suspensions were processed using the Chromium Next GEM Single Cell Fixed RNA Sample Preparation Kit (10x Genomics, PN-1000414) according to the manufacturer’s guidelines. The samples were fixed for 24 hours at 4°C and stored with Enhancer at -80°C until further processing. Library preparation was performed using the Chromium Single Cell Flex Kit (10x Genomics) following the manufacturer’s protocol (CG000527 Rev F). All samples were incubated for 20 hours at 42°C with an anti-human probe set. Each condition had a unique barcode to allow for multiplexing. After pooling the samples and subsequent washes, three lanes of a 10x Chip Q were loaded with each condition being represented in each final lane/library. Raw sequencing data were processed and de-multiplexed using the Cell Ranger pipeline (10x Genomics, Cellranger v. 9.0.0) with human reference genome refdata-gex-GRCh38-2024-A and Chromium_Human_Transcriptome_Probe_Set_v1.1.0_GRCh38-2024-A.

Downstream analyses, including quality control, normalization, library integration, doublet detection, clustering, and differential gene expression analysis, were performed using Seurat (v4.3.0)) in R. Quality control criteria included the removal of cells with fewer than 200 genes detected, fewer than 500 UMIs or with more than 10% mitochondrial gene content. To remove doublets, cells with a UMI count two standard deviations (above the mean were excluded. Further, doublets were identified by mixed gene expression profiles and their characteristic floating and small cluster shape. Harmony(Korsunsky et al., 2019) (v1.2.3) was used for integration of the three batches/libraries. Cell types were annotated based on the top differentially expressed genes of the Seurat clusters. The homeostatic gene sets and neuronal remodeling genes were derived from the current microglia nomenclature consensus(Paolicelli et al., 2022) and transcriptional phenotypes found in literature(Holtman et al., 2017). The environment-dependent signature (*ex vivo* induced) is based on the findings from Gosselin et al., (2017). AD pathology and lipid processing related gene sets are derived from a recent study using post-mortem brain tissue about human microglial state dynamics in AD(Sun et al., 2023). To predict ligand-receptor pairs based on the single cell transcriptomes of our co-maturation cultures and to infer cell-cell communication, CellChat (v2.1.2) was used(Jin et al., 2025). We used the human CellChatDB in default settings and filtered out interactions that had less than 10 cells.

#### Micro-electrode array recording and analysis

Neuronal network activity was recorded using the Axion Maestro Pro system equipped with AxIS Navigator software (Axion Biosystems). On DIV0 hiPSCs were plated on CytoView 48-well micro-electrode arrays (MEAs) and then differentiation was initiated. To ensure stable temperature of 37°C and 5% CO_2_ during measurements, all plates acclimatized for a minimum of 10 min before starting the recording. Recordings were always performed ∼24 hours after medium change as this procedure influences firing. Spikes were detected when voltage deflections exceeded an adaptive threshold of ±6 standard deviations from the noise level. Processing of the MEA data was done using the Axion Biosystems NeuralMetric Tool. Bursts were detected when at least 5 consecutive spikes had a maximum inter-spike interval of 100 ms each. The envelope network burst detection algorithm of was used with these settings: threshold = 1.5, minimum inter burst interval = 100 ms, minimum number of active electrodes = 70%, burst inclusion = 75%.

#### Whole-cell patch clamp recordings

Coverslips were placed in a recording chamber on a microscope stage and were perfused with oxygenated (95% O_2_ /5% CO_2_) artificial cerebrospinal fluid (ACSF) at 32°C. ACSF contained (in mM): 24 NaCl, 1.25 NaH_2_PO_4_, 3 KCl, 26 NaHCO_3_, 11 glucose, 2 CaCl_2_ and 1 MgCl_2_. Pipettes for patch clamp recordings were pulled from borosilicate glass capillaries with filament and fire-polished ends (inner diameter 0.86 mm, outer diameter 1.05 mm, resistance 5–8 MΩ, Science Products GmbH) using a micropipette puller (PC-10, Narishige). For recording miniature excitatory postsynaptic currents (mEPSCs) and AMPA receptor-mediated currents, pipettes were filled with a filtered intracellular solution containing (in mM): 30 potassium gluconate, 5 KCl, 10 HEPES, 2.5 MgCl_2_, 4 Na_2_-ATP, 0.4 Na_2_-GTP, 10 Na_2_-phosphocreatine and 0.6 EGTA. The pH was adjusted to 7.2. Osmolarity was adjusted to 290 mOsmol using a Semi-Micro Osmometer (K-7400, Knauer). Recordings were carried out in voltage-clamp mode, acquired using a digitizer (1140A, Digidata) and an amplifier (Multiclamp, 700B, Molecular Devices), with a sampling rate at 20 kHz and a lowpass filter at 1 kHz. Recordings were not corrected for liquid junction potential (−15.646 mV). Recordings were not analyzed further if series resistance was >25 MΩ or when it became >10% of the membrane resistance. mEPSCs were recorded for 10 min in the presence of 1 μM tetrodotoxin (Tocris, 1069) and 100 μM picrotoxin (Tocris, 1128) at a holding potential of -60 mV at DIV 49. To measure light-evoked synaptic currents, we sparsely transduced neurons with a lentiviral vector containing channelrhodopsin-2 (ChR2) tagged with an enhanced yellow fluorescent protein (eYFP) under a human Synapsin I promoter (Addgene, 20945) at DIV 1. At DIV 28, before patching a neuron, an illumination of 470 nm LED light (KSL2, Rapp OptoElectronic) was given to verify whether the neuron was positive or negative for ChR2. A ChR2-negative cell was recorded in voltage clamp mode at a holding potential of -60 mV and +40 mV. Light-evoked synaptic responses were induced by exciting nearby ChR2-positive cells using a 5 ms illumination with 470 nm LED light through the objective lens (60x). For the final traces, 30 sweeps were averaged. Light-evoked synaptic responses were 2,3-Dioxo-6-nitro-1,2,3,4-tetrahydrobenzo[*f*]quinoxaline-7-sulfonamide (NBQX)-sensitive (50 μM; dissolved in DMSO; Tocris, 0373) indicating these currents were AMPA receptor-mediated (Figure S4). In case a ChR2-positive cell was recorded, the location of the stimulus was adjusted to avoid excitation of the patched neurons.

### Quantification and statistical analysis

Statistical analyses were performed with R (RStudio, R version 4.2.0) or GraphPad PRISM 8.0.0 (GraphPad Software, Inc., CA, USA). All values are reported as mean ± standard error of the mean (SEM), unless stated otherwise. In all figures, P-values are indicated as follows: ^∗^*p* <0.05, ^∗∗^ *p* <0.005, ^∗∗∗^*p* <0.0005, ^∗∗∗∗^ *p* <0.0001. For comparisons between two conditions at one time point, an unpaired t-test was performed. For comparison of two or more conditions with repeated measures, we used repeated measures two-way ANOVA. To detect if neuronal activity metrics show significant monotonic trends across development (DIVs), we applied a Mann-Kendall test. Any time multiple conditions or time points were compared, we applied post-hoc Bonferroni or Sidak correction. If not specified otherwise, the annotation ‘N’ refers to independent replicates defined by measurements in different experiments performed on separate occasions. The annotation ‘n’ refers to technical replicates and is defined per experiment in the figure legends.

## Supplemental information

Document S1. Figures S1–S8

Table S1. Troubleshooting tips for successful co-culture of microglia and neurons

Table S2. Transcriptomic and electrophysiological data

Document S2. Article plus supplemental information
