# Supplementary material for: Development and validation of a long-term co-maturation protocol for human stem cell-derived microglia and neuronal networks
Source: Stem Cell Reports. 2026 Aug 20;21(9):103053. doi: 10.1016/j.stemcr.2026.103053 (PMC13555563; doi:10.1016/j.stemcr.2026.103053)
Supplement: Supplementary file 1 [file mmc1.pdf]

Stem Cell Reports, Volume *21*

Supplemental Information

Development and validation of a long-term co-maturation protocol for human stem cell-derived microglia and neuronal networks

Annika Mordelt, Imke M.E. Schuurmans, Nicky Scheefhals, Marina P. Hommersom, Koen Slottje, Kimberly Mast, Mara Graziani, Carlos O. González, Klaas W. Mulder, Laura J.A. Wingens, Dirk Schubert, Nael Nadif Kasri, and Lot D. de Witte

HiPSC line/clone used for iMGs	Researcher	Lab	Astro	Network activity	ICC staining	Representative image	DIV of image
AICS-0016, cl.18, Allen Institute	AM	LDW	rodent	yes	MAP2 (neurons) and GFP-actin (iMG)	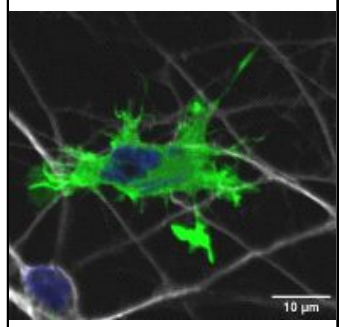	28
GM25256 Coriell Institute	AM	LDW	human	yes	MAP2 (neurons) and TREM2 (iMG)	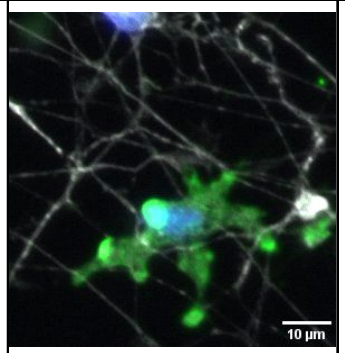	28
HPSI0114i-kolf_2, Wellcome Trust, Sanger Institute	AM	LDW	rodent	yes	MAP2 (neurons) and CX3CR1 (iMG)	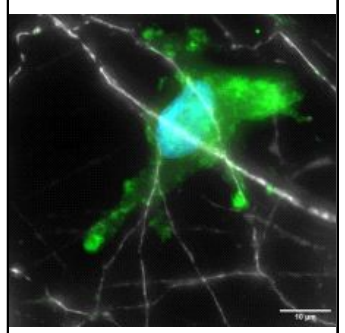	28
GM25256 Coriell Institute	NS	NNK	human	yes	MAP2 (neurons) and NMDAR (iMG)	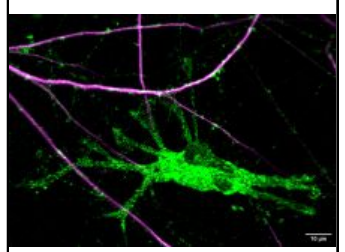	42
GM25256 Coriell Institute	NS	NNK	human	yes	MAP2 (neurons) and brightfield (iMG)	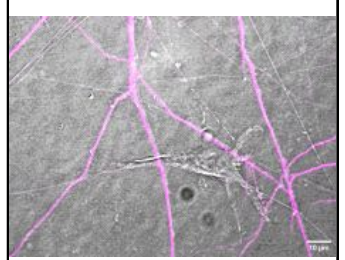	51
GM25256-Crispr/Cas edited clone 1	MG	LDW	rodent	yes	MAP2 (neurons) and IBA1 (iMG)	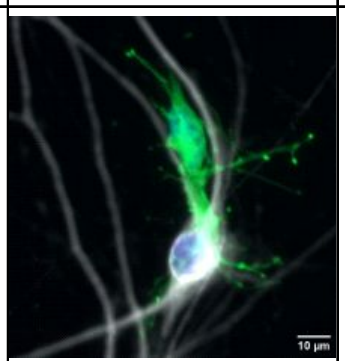	28
GM25256-Crispr/Cas edited clone 2	MG	LDW	rodent	yes	MAP2 (neurons) and IBA1 (iMG)	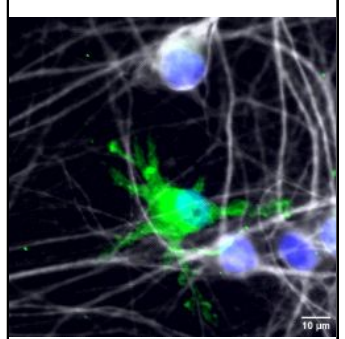	28
GM25256-Crispr/Cas edited clone 3	MG	LDW	rodent	yes	MAP2 (neurons) and IBA1 (iMG)	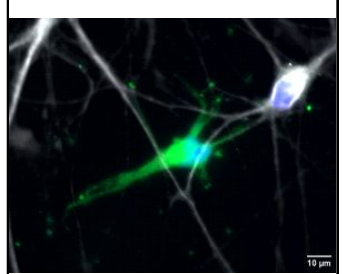	28
GM25256 Coriell Institute	MG	LDW	rodent	yes	MAP2 (neurons) and IBA1 (iMG)	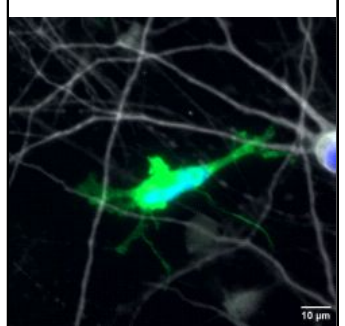	28

Supplementary figure 1. HiPSC lines tested for iMG differentiation in co-maturation protocol by different researchers. Astro = Astrocytes, ICC = Immunocytochemistry, scale bars = 10 µm.

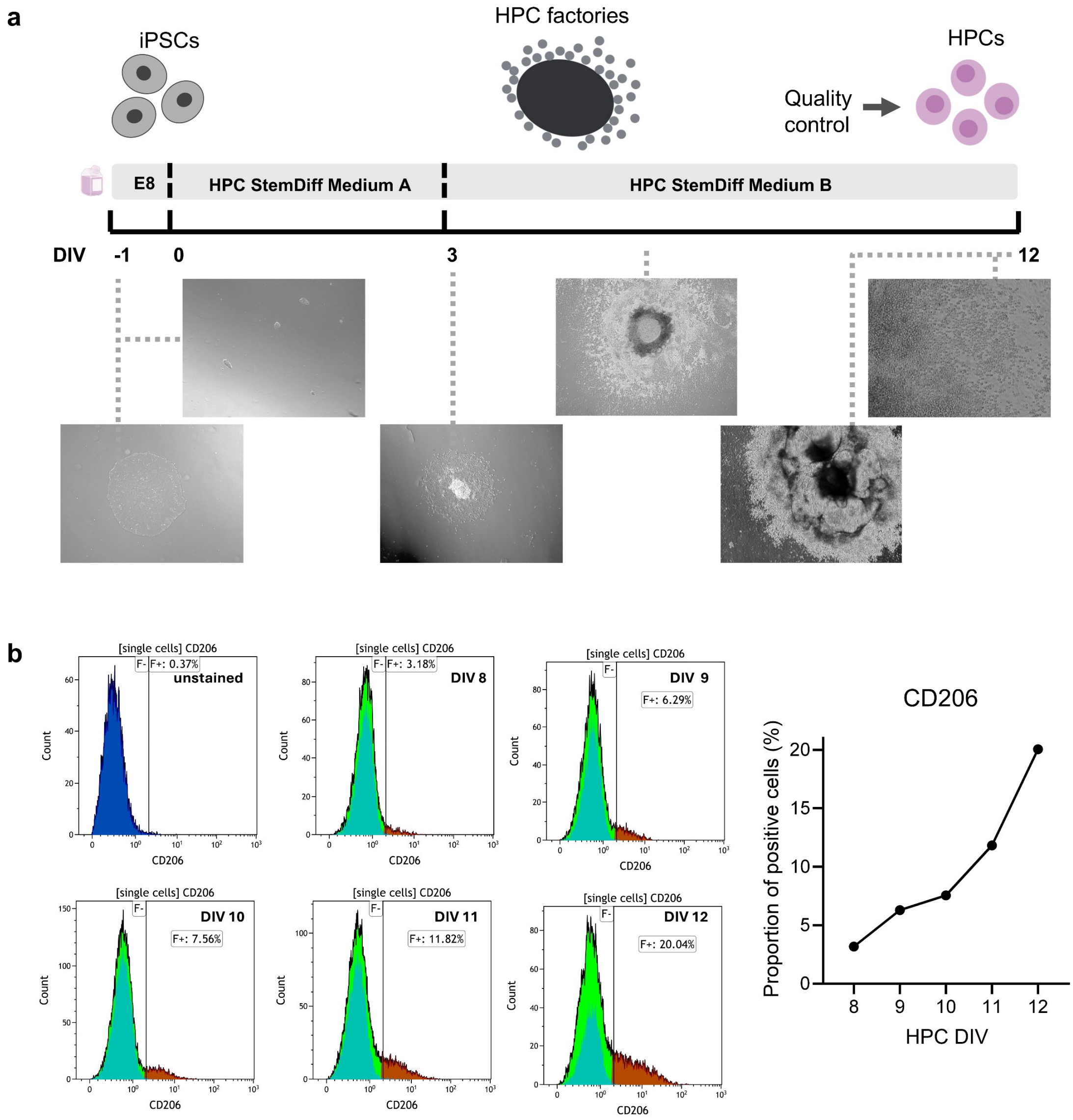

Supplementary figure 2. Timeline of HPC differentiation.

(a) Schematic protocol overview for generation of HPCs from hiPSCs with representative brightfield images of the cultures. **(b)** Flow cytometry analysis of CD206 expression in HPC population sampled at DIV 8 to DIV 12 along the differentiation timeline, N = 1.

a

Neuron-compatible medium

Component	Concentration
DMEM/F12	-
Glutamax	1x
B27	1x
Primocin	100 ug/ml
IL-34	*100 ng/ml*
TGF-β	*50 ng/ml*
M-CSF	*25 ng/ml*

Original McQuade medium

Component	Concentration
DMEM/F12	-
Glutamax	1x
B27	2x
N2	0.5x
NEAAs	1x
Monothioglyerol	400 uM
ITS	1x
Human insulin	4 ug/ml
IL-34	*100 ng/ml*
TGF-β	*50 ng/ml*
M-CSF	*25 ng/ml*

b

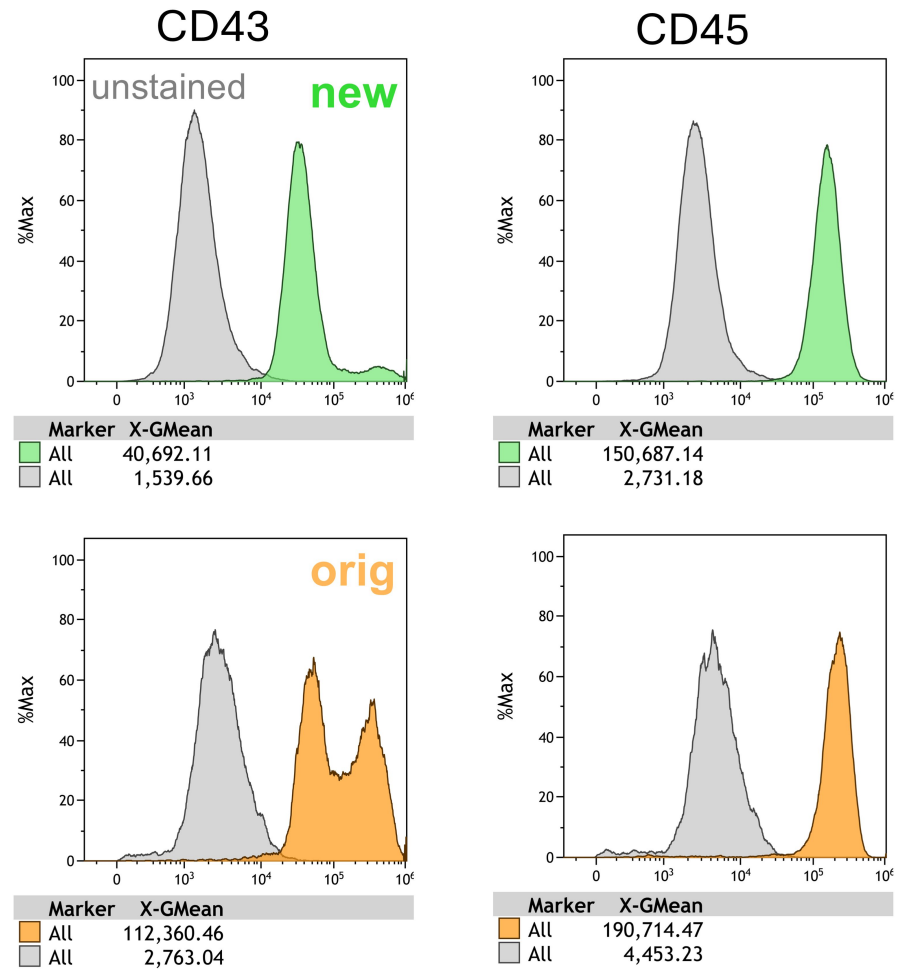

c

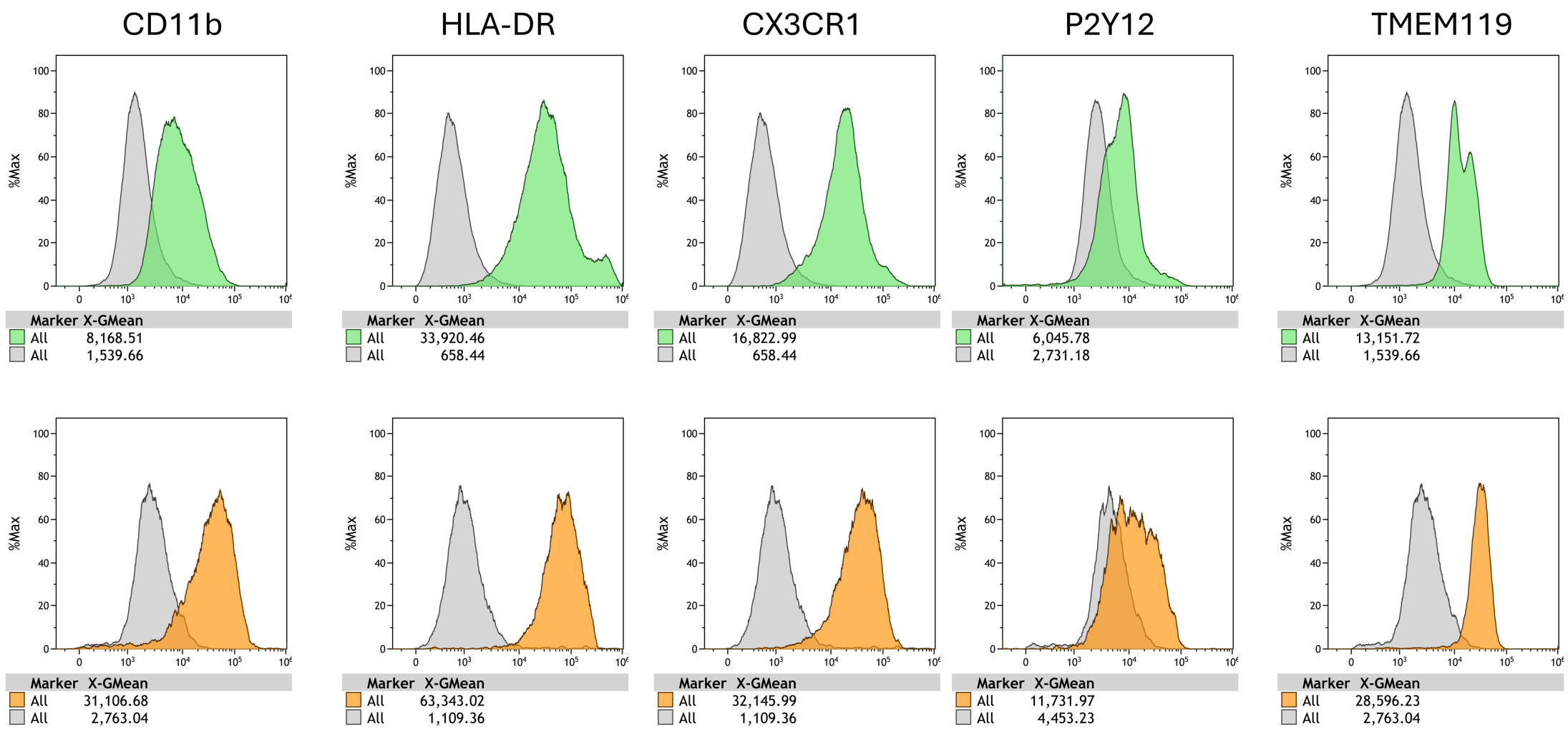

d

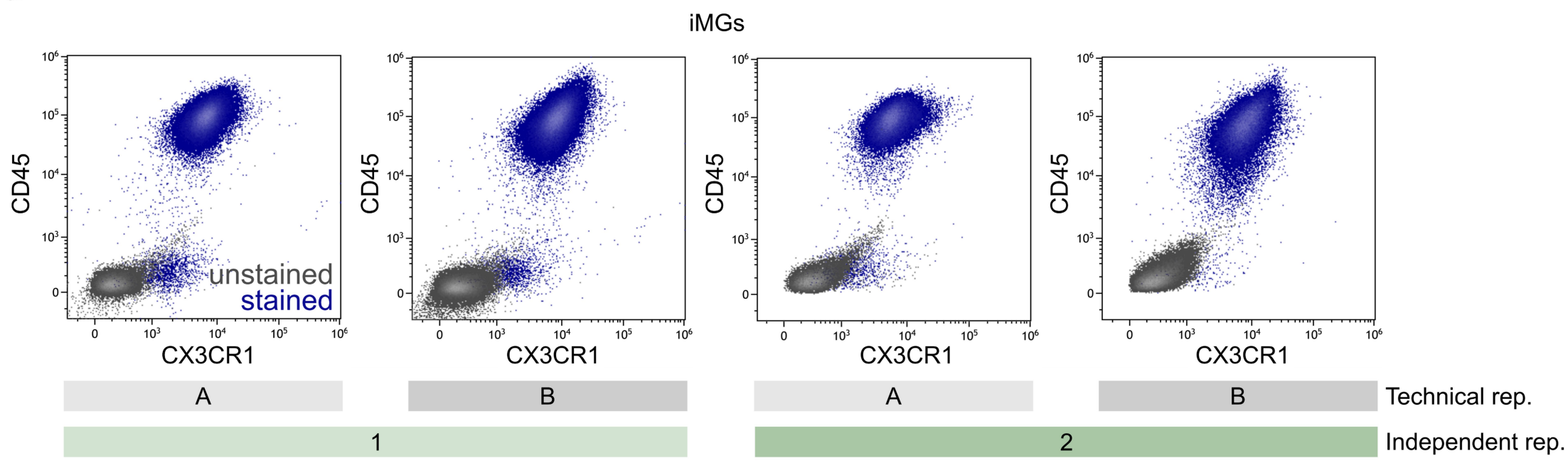

Supplementary figure 3. Comparison original McQuade medium to minimal essential medium. (a) Detailed medium composition of the neuron-compatible and the original medium for iMG differentiation **(b)** Representative Flow Cytometry plots of key myeloid and microglial markers in iMGs differentiated with the two media **(c)** Flow cytometry plots showing consistent expression of CD45 and CXC3CR1 across technical and independent replicates in iMGs differentiated with the neuron-compatible medium.

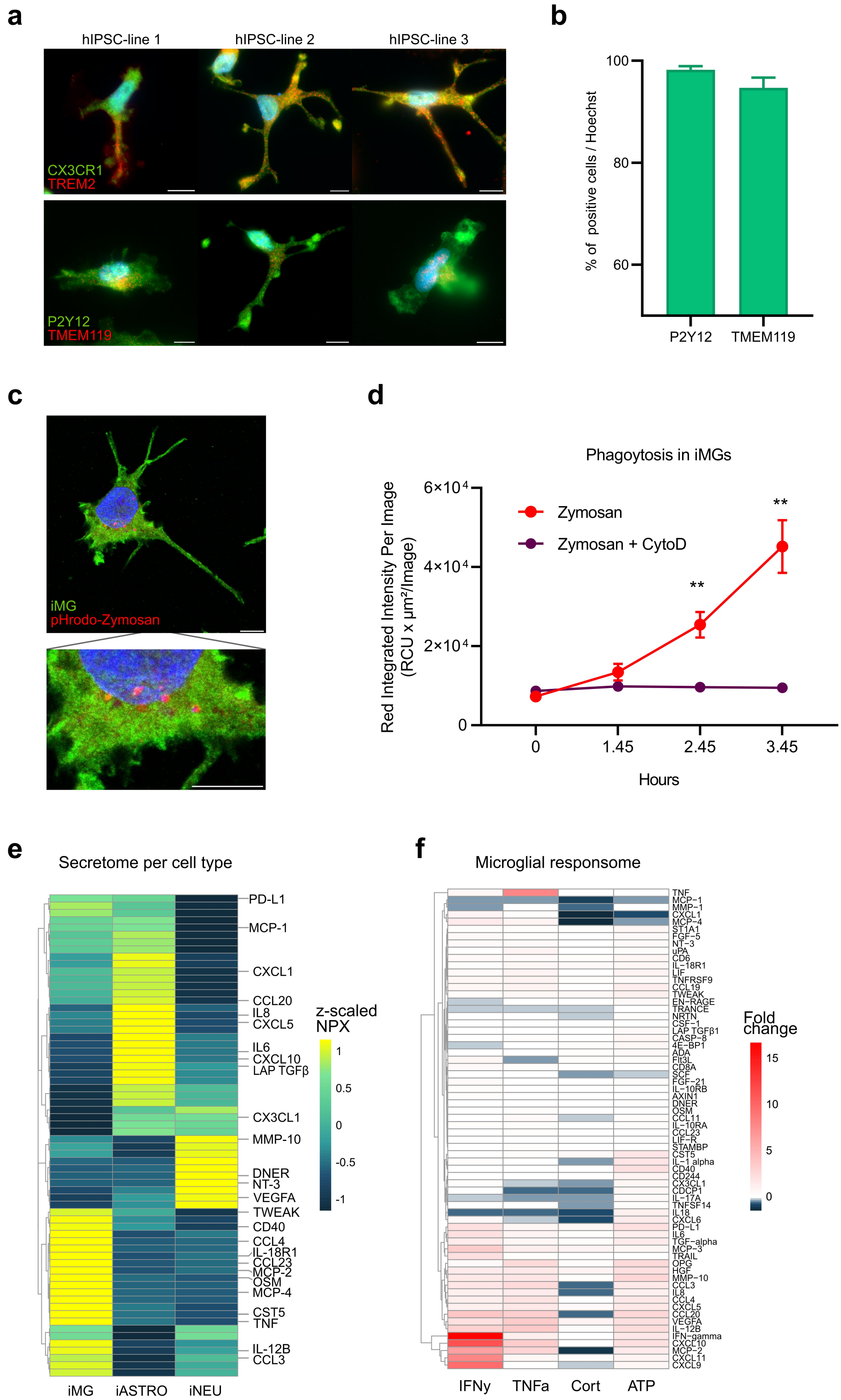

Supplementary figure 4. Assessment of phagocytosis and cytokine production in response to stimuli of iMGs differentiated with neuron-compatible medium.

(a) Representative fluorescence microscopy images of CX3CR1, TREM2, P2RY12, and TMEM119 expression in iMGs at DIV 23, scale bars = 10 µm, hiPSC-line 1 = GM25256, hiPSC-line 2 = HPSI0114i-kolf_2, hiPSC-line 3 = HPSI0314i-hoik_1. **(b)** Quantification of percentage of iMGs expressing key microglial markers in cultures based on immunocytochemistry, n = 17 images for P2Y12, n = 24 images

for TMEM119. **(c)** Representative fluorescence microscopy image of pHrodo-labelled Zymosan bioparticles phagocytosed by iMG at DIV 23, scale bars = 10 µm. **(d)** Quantification of red fluorescence as a proxy for phagocytosis using live cell imaging, n = 4 wells for Zymosan, n = 2 wells for Zymosan + Cytochalasin D (CytoD), two-way ANOVA with post-hoc Sidak correction. **(e)** Heatmap of secreted protein profile by iMGs (MG), astrocytes (ASTRO) and neurons (NEU), measured in monocultures, NPX values are z-scored, N = 1, n = 3 cultures per cell type. **(f)** Heatmap of iMG secretome responses to hydrocortisone (CORT), ATP, IFNγ, and TNFα, log2 fold change normalized to untreated cells is shown, N = 1, n = 2 cultures per cell type. If not otherwise specified, iMGs for data in this figure were generated from GM25256 (Coriell Institute) and AICS-0016, cl.18 (Allen Institute), iNeurons were generated from GM25256.

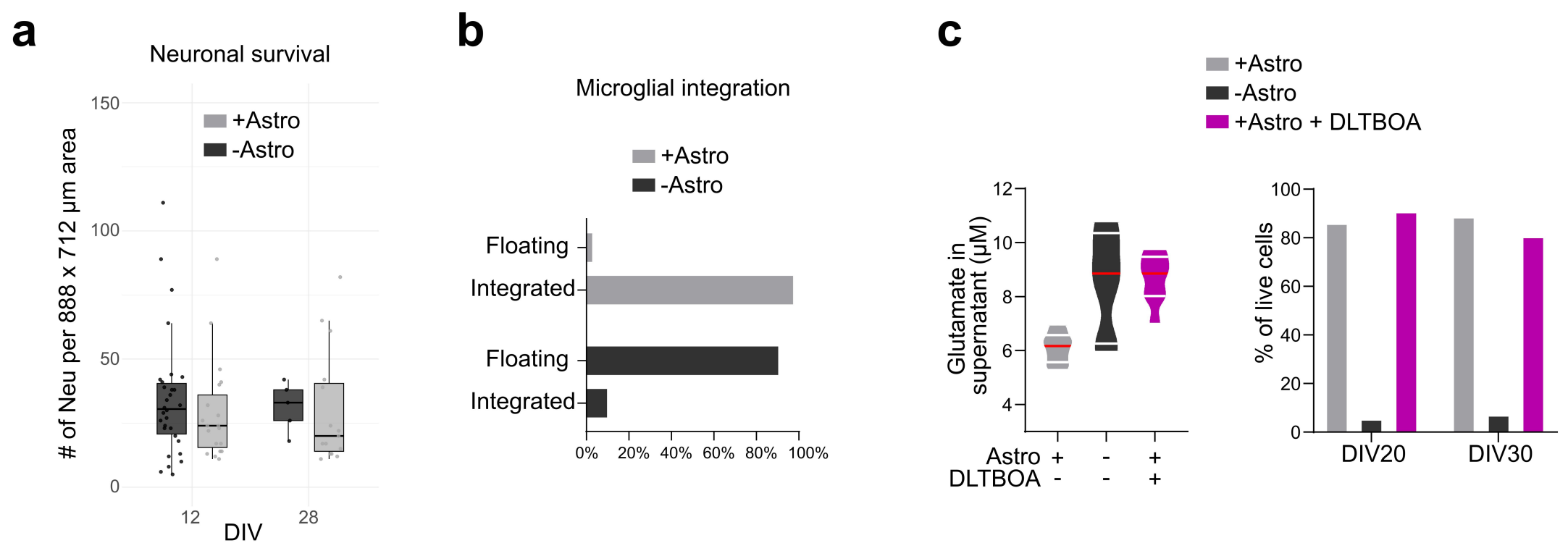

Supplementary figure 5. Astrocytes are required for both microglial survival and integration and this is not dependent on glutamate clearance by astrocytes

(a) Number of neurons per 20x microscopy image in co-cultures with or without astrocytes quantified at DIV 12 and DIV 28, N = 2, n = 34 images for +Astro, n = 36 images for -Astro. **(b)** Percentage of microglia integrated in culture vs floating in supernatant at DIV 20 measured by flow cytometry, N = 1. **(c)** Concentration of Glutamate (µM) in supernatant of cultures across DIV 14 – DIV 20, N = 1, n = 2 measurements per timepoint at 4 timepoints (left) and the percentage of live cells in tricultures measured by flow cytometry at DIV 20 and 30, N = 1 (right).

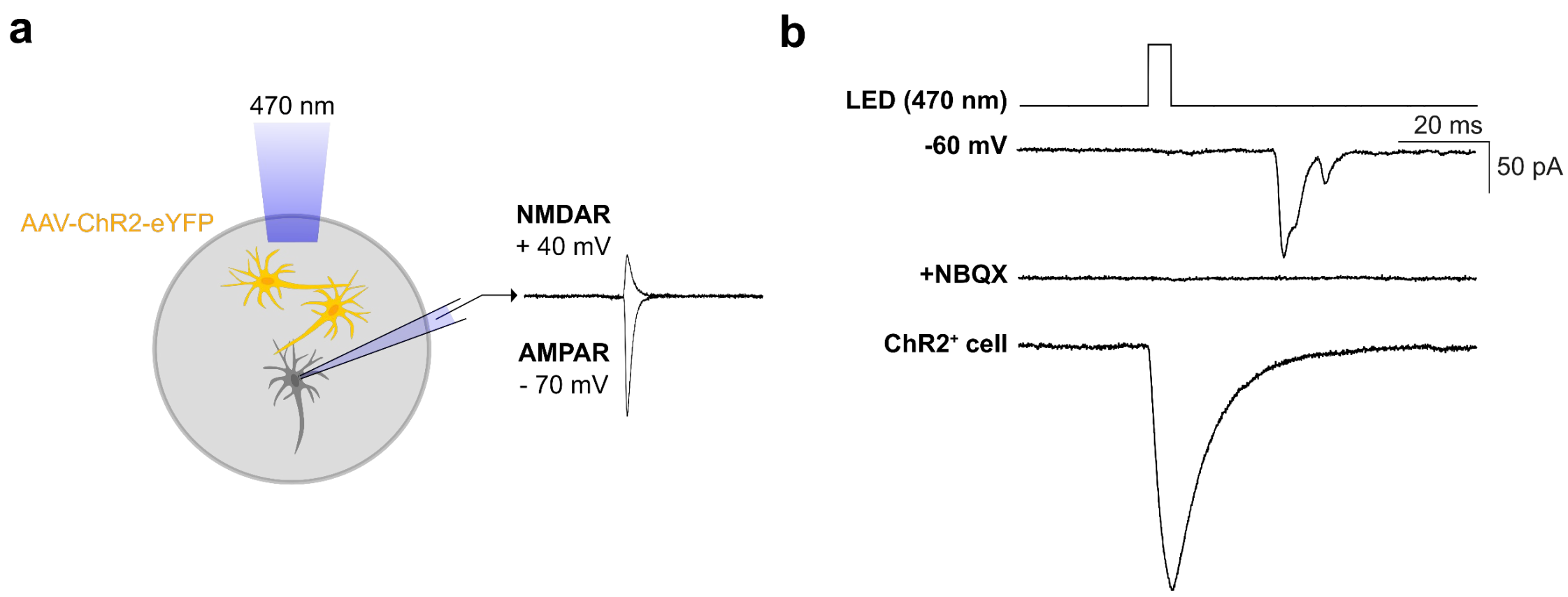

Supplementary figure 6. Patch clamp experiment for optogenetically induced synaptic responses.

(a) Schematic overview of experimental design for optogenetics to induces light-evoked synaptic responses using Channelrhodopsin (ChR2). **(b)** Representative traces of optogenetically induced synaptic response showing no response when NBQX was washed in.

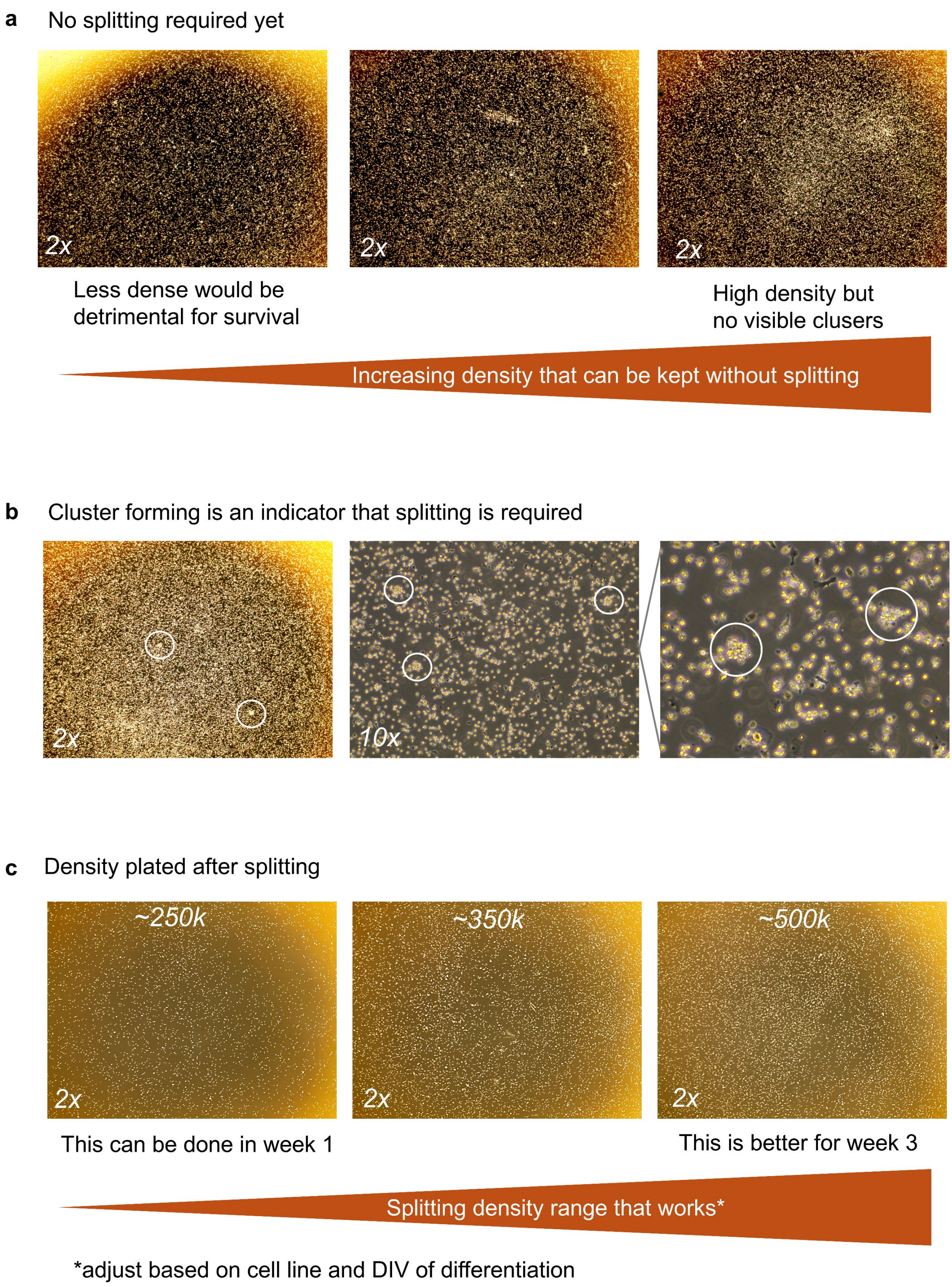

Supplementary figure 7. Recommendations on splitting iMGs.
Representative brightfield microscope images (2x magnification) of iMGs **(a)** that do not require splitting yet, **(b)** forming clusters as an indicator that splitting is required and **(c)** after splitting.

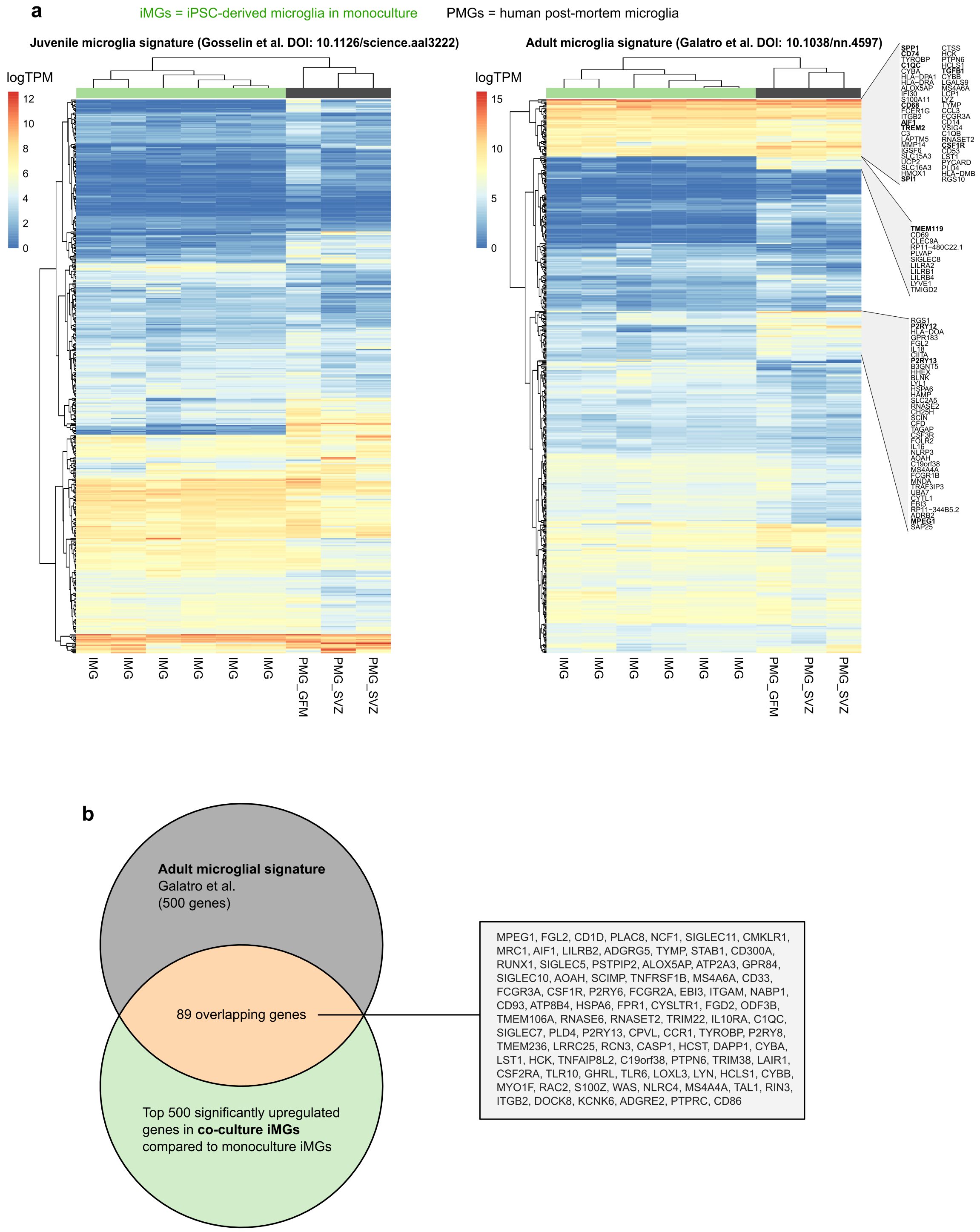

Supplementary figure 8. Microglial gene signatures from public datasets in iPSC-derived microglia.
(a) Heatmaps showing gene expression of 500 microglial genes per signature depicted as log-transformed transcripts per million (TPM), iPSC-derived microglia (iMGs) and post-mortem microglia (PMG) were sequenced in the same batch. GFM = middle frontal gyrus , SVZ = subventricular zone, N = 6 differentiations for iMGs, N = 3 donors for PMGs. Juvenile signature derived from Gosselin et al. (DOI: 10.1126/science.aal3222), Adult signature derived from Galatro et al. (DOI: 10.1038/nn.4597).
(b) Overlap of adult microglial signature with the top 500 significantly upregulated genes in co-culture iMGs compared to monoculture iMGs based on single-cell RNA sequencing data with the 89 shared genes annotated.
